# Parameter-optimized generative adversarial network framework for synthetic MRI generation: fine-tuning critical variables for enhanced image fidelity

**DOI:** 10.3389/fmed.2025.1731370

**Published:** 2026-02-03

**Authors:** Anto Lourdu Xavier Raj Arockia Selvarathinam, Naveenkumar Anbalagan, Parvathaneni Naga Srinivasu, Jaeyoung Choi, Muhammad Fazal Ijaz

**Affiliations:** 1 Department of Data Science and Analytics, College of Computing, Grand Valley State University, Allendale, MI, United States; 2 Department of Information Technology, Sona College of Technology, Salem, Tamil Nadu, India; 3 Amrita School of Computing, Amrita Vishwa Vidyapeetham, Amaravati, Andhra Pradesh, India; 4 School of Computing, Gachon University, Seongnam-si, Republic of Korea; 5 Centre for Artificial Intelligence Research and Optimization (AIRO), Design and Creative Technology, Torrens University Australia, Melbourne, VIC, Australia

**Keywords:** conditional GAN, generative adversarial networks, medical image synthesis, mustGAN, parameter optimization, StyleGAN2, synthetic MRI generation

## Abstract

**Introduction:**

The availability of large-scale medical imaging datasets is often constrained by privacy regulations, high acquisition costs, and ethical concerns. Synthetic medical image generation using generative adversarial networks (GANs) offers a promising solution to overcome these limitations. This study investigates the effectiveness of a Parameter-Optimized Generative Adversarial Network (POP-GAN) and compares its performance with state-of-the-art architectures, including StyleGAN2, multi-stream GAN (mustGAN), and Conditional GAN (cGAN), for realistic MRI image synthesis.

**Methods:**

The proposed framework integrates progressive growing strategies with optimized hyperparameters, including a batch size of 256, learning rate of 1 × 10^−4^, dropout rate of 0.3, and a buffer size of 6,000. All models were trained to generate MRI images at a resolution of 128 × 128. Performance was evaluated using quantitative metrics such as Mean Squared Error (MSE), Mean Absolute Error (MAE), Peak Signal-to-Noise Ratio (PSNR), and Fréchet Inception Distance (FID), along with expert-based clinical realism scoring.

**Results:**

POP-GAN demonstrated a 27% reduction in MSE compared with the baseline model (from 6.58 × 10^−3^ to 4.81 × 10^−3^), achieved higher PSNR, and reduced FID from 32.91 to 24.36. cGAN achieved the lowest MAE (3.50 × 10^−3^), indicating superior reconstruction accuracy. mustGAN produced the strongest resolution fidelity, while StyleGAN2 delivered the highest perceptual realism. POP-GAN also attained a clinical realism score of 4.13 out of 5.

**Discussion:**

The results demonstrate that parameter optimization and progressive training substantially enhance synthetic MRI quality. POP-GAN provides a balanced trade-off between reconstruction accuracy, perceptual realism, and clinical relevance, supporting its potential for privacy-preserving dataset augmentation and robust medical imaging research.

## 1 Introduction

Modern healthcare depends fundamentally on medical imaging because it provides non-invasive methods to see inside the body, which leads to both correct diagnoses and treatment strategies. MRI demonstrates superiority as an imaging modality because it delivers exceptional tissue contrast alongside no requirement for ionizing radiation. MRI healthcare requires the solution of multiple difficulties because expensive equipment takes lengthy periods to complete scans, and hospitals struggle to obtain them because privacy rules limit data access.

The research fundamentals are anchored in the severe lack of various, annotated datasets of MRI through privacy-related issues, such as HIPAA/GDPR, their high cost, and the lack of ethical standards, which impede the development of effective AI-based diagnostic models. The current GAN models are known to experience unstable training and sub-optimal fidelity in the low-density regime, which creates artifacts that restrict their use in clinical environments. This study solves these problems by proposing a POP-GAN that trains hyperparameters specialized to the MRI properties so that an image becomes more realistic and varied. This allows augmenting datasets for rare diseases, 2-10% faster clinical algorithm training using the prior research (1–5), and protecting patient privacy, which has considerable advantages compared to generic GANs in resource-limited healthcare.

The deep learning revolution in medical image analysis involves enormous datasets for achieving optimal results, but this approach emerged only recently. The shortage of properly annotated medical imaging databases functions as the primary restricting factor. The shortage of suitable medical data has triggered heightened interest in artificial data creation methods, and GANs stand out as the most promising solution. Medical images produced by GAN systems demonstrate exceptional capability to create realistic medical data, which helps increase the size of available datasets and develops algorithms in addition to serving as a teaching tool without endangering patient privacy.

In 2014, Goodfellow et al. (1) developed GANs, which contain two competing networks for data synthesis, where one network generates new data while the other network differentiates true data from synthetic results. The adversarial process between the two networks drives the generator toward producing more real-looking images throughout training. The medical field applies GANs across multiple imaging modalities, such as MRI, computed tomography (CT), and ultrasound, to perform data augmentation tasks and generate synthetic data between different modalities.

Recently, the development potential of GANs in the field of image analysis of medicine and agriculture has caused a paradigm shift with regard to the problem of data shortage and diagnostic accuracy. Chong and Ho (2) showed that GANs could create realistic 3D MRIs of the brain that included matching anatomical texture, allowing data augmentation of neurological research. Jiang et al. (3) introduced a Wasserstein GAN-based O-Net network in neuroimaging that enabled accurate extraction of brain regions within the MRI imaging modality, showing robustness across a wide range of datasets. Congesting the medical field, Wang and Xiao (4) also utilized GAN-generated synthetic data to enhance the accuracy of defect detection on lychees in agricultural products by 12-15%. Such advancements have found their way to dental diagnostics, as SegAN was suggested by Srinivasu et al. (5) to predict the presence of caries on the basis of 2D-panoramic X-rays with 93% segmentation precision, and without endangering the patient's privacy. All in all, these studies highlight how GANs have the potential to transform the limitations of data and have the ability to improve analytical accuracy across fields.

Brain MRI applications using GANs attract growing research interest because they support disease status assessment, tumor detection, and brain structure investigations. Research indicates that GAN-generated synthetic MRI data improve medical diagnosis of neurological conditions by 2–10% across different disorders through data augmentation techniques. Medical imaging practitioners demonstrate the particular usefulness of GAN-based data augmentation when working with rare or unbalanced conditions.

Current challenges exist in the generation of high-fidelity medical images based on GAN models, where clinically valid results need to exist alongside anatomical precision. Many existing approaches face problems with unnatural tissue marking, along with artifacts from fluid-attenuated inversion recovery (FLAIR) sequences, and insufficient pathological representation and insufficient paradigm flexibility between acquisition setups. The quality and utility of GAN-generated MRIs depend heavily on how the model architecture and parameters are designed. Suboptimal settings within a GAN generate restricted output varieties while creating unstable training conditions that yield inadequate result features. The existing research on medical image synthesis through GAN architectures still lacks standardized approaches to optimize the GAN parameters for MRI generation.

Medical image collections differ from regular images due to their standardized acquisition procedures combined with homogeneous body structures and distinctive noise characteristics. Unique properties of MRI data demand specialized parameter optimization strategies because they include multiple contrast mechanisms, such as T1, T2, and FLAIR, as well as specific signal-to-noise ratios and required spatial resolution levels. Medical image standardized acquisition protocols guarantee consistency in anatomical structures but incur modality-dependent noise, which can be counteracted by GAN training using accommodating hyperparameters, such as adaptive learning rates and dropout. This noise homogeneity demands progressive learning, which learns fine details over time; otherwise, the generator will overfit to the artifact. The ethical aspects and medical application scope of synthetic images require complete validation mechanisms which expand further than traditional computer vision criteria.

The study bridges the disconnect in GAN theory and practical synthetic MRI generation through an optimized framework which optimizes image quality while maintaining sufficient clinical usefulness. Research investigates generative adversarial networks' ability to change medical imaging through a review of present synthetic MRI generation studies and the identification of existing obstacles. A new Parameter-Optimized Progressive GAN framework named POP-GAN serves to optimize image generation and create clinically relevant synthetic MRI through system-based parameter optimization. The uniqueness of our approach derives from its detailed optimization strategy, which includes evaluating network structures, training methods and loss algorithms, and progressive development protocols, all designed explicitly for MRI image characteristics. Our research utilized three types of validation metrics, such as Fréchet Inception Distance (FID), SSIM, and MSE, to prove our work alongside comparative testing of the fully optimized POP-GAN against GAN baselines. Through a complete comparison, our methodology proves to enhance synthetic image quality along with diversity and practical usage capabilities for potential medical research applications and training, together with solutions for patient data protection. The metrics were chosen due to their complementary capabilities of medical image evaluation: MSE and PSNR assess the accuracy of the reconstruction at the pixel level, which is necessary to preserve the anatomic structure; SSIM is supportive of the medical clinical practice as it assesses the structural similarity; FID, in its turn, evaluates distributional fidelity at the high-level features and therefore is supportive to ensure that the synthetic image is perceptually realistic and diverse.

POP-GAN contrasts with the recent state-of-the-art architectures such as MedFusionGAN, CollaGAN, and U-shaped transformer GAN, which are concerned with multi-modal fusion, collaborative synthesis, and feature learning with transformers. Although these can be of high quality in perceptual understanding, they can need large datasets and computation and are not applicable to private and data-scarce clinical contexts. The significance of the POP-GAN is that it entails a new systematic parameter optimization in MRI due to the noise and contrast characteristics, and achieves stable training with small samples and fewer artifacts; it can be applied in low-resource environments. This supersedes such disadvantages of baselines as Progressive GAN with incremental resolution without domain tuning, and StyleGAN2 is style modulation but with high data requirements, as explained in Table 1, and makes POP-GAN a practical solution to the next stage of developing diagnostic AI and learning tools.

**Table 1:** Comparison of POP-GAN novelty and metrics vs. advanced GAN variants.

**Model**	**Key novelty/features**	**MSE ( × 10^−3^)**	**PSNR (dB)**	**SSIM**	**FID**	**Strengths in medical context**
Progressive GAN	Incremental resolution growth; no MRI-specific tuning	~5.50	~22.00	0.85	~28.00	General resolution scaling; prone to instability in sparse data
StyleGAN2	Style-based modulation; path length regularization	4.2	28.6	0.9	18.4	High perceptual quality; resource-intensive for medical sparsity
mustGAN	Multi-stream for multi-contrast synthesis	3.9	32.1	0.885	20.15	Excellent resolution; complex for single-modality MRI
cGAN	Conditioned generation for targeted outputs	4.5	26.4	0.87	22.8	High reconstruction accuracy; limited diversity without optimization
POP-GAN (proposed)	MRI-specific parameter optimization + progressive growth	4.81	23.18	0.891	24.36	Balanced stability in sparse data; lower MSE vs. baseline (~27% improvement); clinically realistic

POP-GAN is technically novel, featuring systematic, MRI-specific hyperparameter optimization and a progressive growth protocol, which increases training and image fidelity in data-limited medical conditions. In contrast to standard Progressive GAN, with paramount emphasis on incremental resolution with no domain-specific fine-tuning, POP-GAN applies parameter adjustment to MRI properties, such as tissue contrast or noise structures, which decrease mode collapse and artifacts. POP-GAN is more sparsity stable than StyleGAN2, which, despite being capable of high fidelity through style modulation as well as requiring large datasets, demonstrates a higher perceptual quality in comparison with POP-GAN. This leads to strong performance across multiple evaluation measures, as summarized in Table 1, where approximate values are marked with the symbol (~).

On the other hand, StyleGAN2, which favors perceptual quality with style-based modulation, and POP-GAN, which includes a systematic parameter optimization to enhance stability when operating with sparse data. In comparison, POP-GAN uses less computing resources than mustGAN, which is a multi-stream architecture that focuses on resolution augmentation. POP-GAN uses progressive training together with MRI-specific hyperparameter optimization, resulting in lower MSE, greater PSNR and better clinical realism. This is more efficient and repeatable in real-world medical image generation than Progressive GAN, which builds up resolution incrementally.

This study provides essential solutions to the urgent requirement for top-quality synthetic MRI datasets because these resources advance multiple applications such as algorithm building, clinical system education, and data expansion for scarce medical cases. The optimization effort for GAN parameters seeks to develop a framework that medical professionals can use across different clinical applications and implement into their routines.

The main conceptual contribution of this study is that it presents a parameter optimization framework in GAN-based medical image generation, adjusted to the specific demands of MRI data. In contrast to previous models, which use generic hyperparameter optimization or one-size-fits-all designs, POP-GAN proposes the following main innovations. POP-GAN sets a controlled benchmark to measure the impacts of the batch size, learning rate, dropout, buffer size, and activation slope on MRI synthesis. This systematic tuning finds the best settings to stabilize GAN training, improve convergence, and maximize image fidelity, with limited and heterogeneous medical imaging datasets. POP-GAN also optimizes progressive growth of resolutions based on the MRI-specific characteristics of textures in the anatomy, contrast levels, and noise patterns. This customization can provide anatomically reliable image results and minimize typical artifacts, such as fake tissue delimitation, creating a valid clinical, high-resolution synthetic MRI applicable in diagnostic, educational, and research applications.

The framework also shows good results using small training samples, which solves the biggest problem of medical imaging data scarcity. POP-GAN is stable in training, does not collapse into a single mode, and produces a wide range of clinically plausible synthetic MRI, which makes it suitable to be used in rare disease research and low-resource healthcare research settings. POP-GAN is tested on a multi-metric framework of FID, SSIM, MSE, PSNR, and clinical realism score (CRS). This overall review will guarantee that synthetic images are not only visually persuasive but also clinically significant, giving future researchers a repeatable pipeline to understand the quality and applicability of synthetic images in a real-world clinical setting. Collectively, these developments offer a community-ready, reproducible, standardized framework that will bridge the knowledge gap between the theory of GAN and clinically relevant MRI synthesis. POP-GAN is therefore a contribution to concept-to-clinic, rendering synthetic data generation reliable, scalable, and applicable in clinical-world medical AI pipelines. The key contributions of this research are as follows:

- Proposes a process of optimizing key GAN hyperparameters like batch size, learning rate, dropout, buffer size, and Leaky ReLU slope that can be explicitly used in MRI synthesis, resulting in higher-quality images and a more stable training.
- Introduces a progressive GAN model with optimal values of parameters showing better results than the baseline methods in a synthetic MRI generation that reported lower error and increased perceptual scores.
- Comparisons of the top GAN architectures (StyleGAN2, mustGAN, cGAN) optimized in the context of generating MRI and identifying the relative strengths of each model with respect to resolution, perceptual quality, and precision of reconstruction.
- Shows consistent production of virtual medical images to support the growth of datasets, patient privacy, and clinical algorithm development.

Key research gaps include the lack of standardized, MRI-specific parameter optimization in GANs, leading to unstable training, mode collapse, and poor clinical fidelity in sparse datasets. This study bridges these by proposing POP-GAN, with main contributions: (1) A novel framework optimizing hyperparameters with batch size of 256, learning rate of 1 × 10^−4^ for enhanced stability and fidelity; (2) Progressive growth customized to MRI textures, reducing MSE by approximately 27% compared with baseline models; (3) Comprehensive benchmarking against StyleGAN2, mustGAN, and cGAN, identifying strengths in resolution, perceptual quality, and reconstruction; and (4) Demonstration of clinical utility via improved CRS (4.13/5) and dataset augmentation for privacy-preserving AI development.

This report is organized as follows: Section 2 gives an overview of the literature review; Section 3 clarifies the Proposed Methodology. Section 4 identifies the Experimentation, Results, and Analysis. The Conclusion and Future Scope are defined in Section 5.

## 2 A literature review

GANs have become potent applications to medical imaging, and they can be used to perform image synthesis, image augmentation, super-resolution, denoising, fusion, and missing sequence reconstruction. Numerous studies indicate that they have the potential to improve the accuracy of diagnosis, resolve the problem of data scarcity, and maintain patient privacy, yet their clinical validation and application in generalized settings are still challenging.

Chen et al. (6) conducted a review of 105 studies on GAN-based medical image augmentation that focuses on models, datasets, loss functions and metrics that demonstrate that GANs can work with limited data. The study is generally descriptive, although very detailed, and there is no comparative assessment or practical advice that would allow one to directly apply it in real-world medical imaging assignments.

Singh and Raza (7) summarized the use of GANs in medical imaging, mentioning such frameworks as DCGAN, CycleGAN, pix2pix, and UNIT. GANs can be used in image augmentation, reconstruction, registration and cross-modality translation. The enhancement in performance of hybrid architectures is a plus, but these issues must be addressed: clinical validation, realism, and generalization, which is an indication of the future of research in medical image synthesis and analysis.

GANs have a transformative potential in brain imaging, including the ability to segment, synthesize, reconstruct, and model the disease (8). Although the review is well-structured clinically, presents a clear vision of how it can be applied, and provides a glimpse into the future, it is mostly descriptive, not providing the quantitative performance comparison and implementation schemes, which restricts their practical use in the development of real-world neuroradiology processes.

Dar et al. (9) presented a cGAN based on multi-contrast MRI synthesis, where structural details are kept with the help of adversarial, pixel-wise, perceptual, and cycle-consistency losses and with the help of neighboring slices. It has been shown to be superior to the conventional performance in the generation of missing or corrupted contrasts, greater diagnostic usefulness, and higher multi-contrast MRI quality without the need to spend long scan times.

Dimitriadis et al. (10) explored synthetic MRI data obtained by generative models, such as GANs and VAEs, to improve cancer differentiation, which solves the problem of data scarcity, ethical issues, and high expenses of data acquisition. Although exhaustive and advocating open science, the research mostly addresses the methodology and best practices without involving empirical performance comparisons and validation on various clinical datasets.

Noor et al. (11) introduced a two-module GAN system, which is known as DLGAN, to reconstruct undersampled MRI with state-of-the-art PSNR and SSIM, using hierarchical feature extraction and K-space information. Although it minimizes artifacts and increases efficiency, the study does not discuss the generalizability to a variety of MRI protocols and clinical implementation.

According to Sun et al. (12), cGANs could be deployed to create synthetic MRI data of ankylosing spondylitis, ensuring patient privacy, fidelity, and diversity. The research is efficient in terms of discussing the issues of privacy, but it is evaluated with respect to particular clinical images only, and the generalizability and applicability of the synthetic data in the real context remain unclear.

Chen et al. (13) provided a comparison of X-ray, MRI, CT, and ultrasound in the diagnosis of musculoskeletal diseases with strengths, limitations, costs, and accessibility, as well as integrating AI. Although very broad and clinical in its relevance, the study is more of a review of the already existing knowledge rather than offering new data through experimental procedures to help the field of diagnostic methodology or comparisons of the evidence-based performances.

Chlap et al. (14) systematically presented medical image data augmentation methods of CT and MRI and classified them into basic, deformable, and deep learning-based methods. Although the article has done an excellent job of consolidating augmentation strategies and their clinical applicability, it does not provide a quantitative evaluation or comparative analysis of model performance that can be used to provide actionable information regarding the application of the strategy in real-world settings.

Ali et al. (15) conducted a review of 139 articles on the use of GANs in brain MRI, and they noted the following three areas: data augmentation, segmentation, and image translation. Although the review is thorough and rigorously conducted methodologically, the main focus of the review is the presentation of descriptive research results without any performance comparison, which prevents some information about the clinical implementation and the effectiveness of GAN-generated MRI data in real-life settings.

Ghassemi et al. (16) suggested that a deep neural network can be pre-trained to serve as a GAN discriminator in extracting features, and then three types of brain tumors can be classified. The technique enhances precision of a 3,064 images dataset of MR. Although it works, because of having a comparatively small dataset, this method might not be generalizable when different clinical situations are considered.

SinGANs generate synthetic prostate MRIs which are of high quality, and Xu et al. (17) verified the quality of synthetic images by means of expert and anomaly detection. Findings show that synthetic images are rather close to actual data. Although it is promising in the field of supervised learning and clinical use, the study has a shortcoming in terms of generalizability, as it was specific to particular trials and may not be applicable to the wider populations of prostate cancer.

The SOUP-GAN is a perceptual loss-based GAN that Zhang et al. (18) proposed to use to focus on 3D MRI super-resolution, creating thinner slices with a better texture, deblurring, and anti-aliases. The approach beats the traditional methods. Although innovative, it has a challenge in generalizing to various modalities and clinical conditions, and more validation is needed to make it a routine clinical use.

Lee et al. (19) introduced a collaborative GAN, CollaGAN, an imputation-based image-to-image multi-domain translation using only one generator-discriminator, which uses multi-domain image-to-image translation. Findings indicate that visual quality is better than that of current techniques. Although effective, the emphasis of evaluation is on visual comparisons, and minimal exploration of clinical applicability or strength across a wide range of medical data has been done.

Yaqub et al. (20) suggested GAN-TL, which is a hybrid approach that combines GANs and transfer learning in reconstructing MRI using under-sampled data. The approach is better in PSNR and SSIM in both brain and knee images using fewer training samples. Although it has potential in clinical scaling, it is only validated in specific organs, and needs more general testing before it can be used widely.

Zhang et al. (21) suggested BPGAN, a 3D U-Net-based GAN to generate PET scans out of MRI to diagnose AD, and it can achieve high MAE, PSNR, and SSIM on Alzheimer's Disease Neuroimaging Initiative (ADNI). Although the synthetic PET did enhance the diagnostic accuracy marginally, the margination was not very high, and hence, further optimization and a clinical validation are required to fully adopt the technology.

Yurt et al. (22) introduced mustGAN, which uses one-to-one and many-to-one synthesis to do multi-contrast MRI, enhancing image quality and diagnostic utility. The technique has better results than state-of-the-art techniques both in quantitative and radiological assessment. Nonetheless, it might be too complicated to be clinically scaled and needs additional validation in a variety of datasets and real imaging cases.

According to Mahapatra et al. (23), an active learning model that can be used to optimize the classification and segmentation of chest X-rays is a Bayesian sample selection with cGAN-generated images of the chest. The approach attains state-of-the-art performance with minimum samples. Even though it is efficient, only particular imaging tasks are studied, and generalizing the results to a wide range of medical applications is necessary.

XCAT-GAN is proposed by Amirrajab et al. (24) to generate 3D consistent and labeled cardiac MRI with anatomically varied XCAT phantoms that enhance the accuracy of segmentation with minimal real data. The method improves Dice scores and minimizes Hausdorff distance. Although it is useful in augmentation, the use of simulated phantoms creates issues of realism and clinical translation.

The concept suggested by Dikici et al. (25) is a narrowed-down GAN ensemble model capable of producing representative and privacy-protected synthetic medical images. Their scale of adaptive ensemble, Frechet distance filtering and validation of mutual information guarantee quality and diversity. Although its performance is similar to that of real data, scalability between modalities and larger cohorts is unexplored.

Ahmad et al. (26) presented a super-resolution model based on GAN, which gradually recovers high-resolution medical images based on low-resolution image inputs by means of multi-path shallow feature extraction, ResNet34 deep features, and residual upscaling. They are more realistic and accurate in more modalities. But there are still certain problems such as computational overhead and lack of validation on clinical outcomes.

Hagiwara et al. (27) used a cGAN to improve the quality of synthetic FLAIR MRI to obtain better PSNR, reduce error, and artifacts than synthetic images at the baseline, and retain lesion contrast. It is an effective study but the cohort used is small (40 MS patients), which makes it difficult to generalize and its wider validation by different pathologies is necessary.

Safari et al. (28) suggested MedFusionGAN, which is an unsupervised GAN that can be used to fuse CT and MRI in order to combine bone and soft-tissue information. It performs better than most traditional and deep learning fusion algorithms on a multicenter brain tumor dataset on structural preservation, contrast, and edge fidelity, but still some of the measures are in the second place, so there is still room to optimize consistency across all the evaluation measures.

Luschi et al. (29) produced the region-specific GAN framework with StyleGAN3-t to produce synthetic malignant melanoma images without involving any specific areas, such as the face, palm, and sole. With a Fréchet Inception Distance (FID) of 31.73, the model generates high-quality diverse images to be used in data augmentation. Nevertheless, it is a future work to clinically validate and compare the architecture with other GANs.

Sharma and Hamarneh (30) introduced a multi-modal GAN that can be used to generate missing MRI pulse sequences based on the existing sequences through a multi-input, multi-output system. The method successfully reconstructs one or more missing sequences with a single pass on brain MRI datasets and has been shown to be better than unimodal and existing multimodal methods. Limitations are dependency on high-quality input sequences and variability that may occur across protocols used to make acquisitions.

Gheorghita et al. (31) generated synthetic cine MRI with GAN to supplement datasets to quantify cardiac function, especially disease phenotypes that are underrepresented. Training a CNN on this synthetic data enhances the forecast of end-diastolic and end-systolic volumes and ejection fraction, and it is better than traditional segmentation techniques. Nevertheless, its applicability to a wide variety of scanners and pathologies is yet to be completely tested.

Safari et al. (28) presented MedFusionGAN, an unsupervised GAN architecture that is used to generate CT-MRI fusions, which merge bone and soft-tissue data, to improve radiotherapy planning. Its performance is assessed on a multicenter dataset of brain tumors, where it is seen to perform better than most traditional and deep learning fusion approaches on six out of nine quantitative metrics, but some metrics are ranked second, meaning that it can be improved slightly.

Chintapalli et al. (32) introduced GenMIND, which is a generative model generating synthetic brain MRI-derived regional volumetric features. It was trained on 40,000 scans of the iSTAGING consortium to produce 18,000 samples that produce real data distributions over 22-90 years. GenMIND enhances downstream disease classification, overcoming privacy and data-sharing limitations, but clinical validation remains to be done.

Wang et al. (33) suggested CSegSynth is a deep generative model that generates specific 3D brain MRI segmentation (white matter (WM), gray matter (GM), Cerebrospinal Fluid (CSF)) in single individuals using just the demographic, cognitive, and interview data without relying on the structural scan itself. This model is more effective than C-VAE, C-GAN, and C-LDM, with low mean absolute volume errors, but the wider applicability to a wide range of populations should be considered.

Kelkar et al. (34) assessed the ability of GANs to learn canonical statistics about medical images, which are useful in objective quality measurement. Although the GAN has demonstrated the ability to reproduce simple first- and second-order statistics as well as generate perceptually realistic images, it does not learn some per-image statistics correctly, and there is a need in medical imaging applications to assess it objectively instead of focusing on visual fidelity.

Logan et al. (35) discussed 3D CNNs and GANs used in the classification of Alzheimer's disease based on multi-modal MRI and PET images. They emphasize the importance of GANs in overcoming the problem of limited data and in the idea of ensemble learning to enhance the robustness of CNN. Though promising, it will require large, heterogeneous datasets which will have to be validated in clinical populations.

Yang et al. (36) presented a GAN-based low-dose CT denoising algorithm based on the Wasserstein distance and perceptual loss to minimize noise at the cost of important structural information. In contrast to the conventional MSE-based techniques, the method does not lose diagnostically significant characteristics and does not deteriorate the image quality, but its effectiveness in relation to different scanners and pathology should be further supported.

The approach offered by Gao et al. (37) is a multi-task transformer model of autism spectrum disorder detection based on rs-fMRI data that involve multiple correlated tasks and an attention system to improve feature extraction and interpretability. The model is more accurate, sensitive, and specific than the state-of-the-art techniques. Nevertheless, it can only be applied to limited cases when it is based on single-modality data and needs larger and more diverse cohorts to be generalized across populations.

Zuo et al. (38) recommended a transformer-based distribution-regularized adversarial graph autoencoder (DAGAE) to produce synthetic brain functional networks to diagnose dementia. The method maintains topological properties through regularization of the latent space and long-range dependencies between nodes, boosting the performance of the classifier to 85.33% on ADNI. Its use of graph representations, however, can make it less applicable to non-connectivity data, and much wider testing on a variety of cognitive disorders and multi-site data is required to validate generalizability and clinical use.

Li et al. (39) introduced ERANet, a semi-supervised meniscus segmentation framework which integrates edge replacement augmentation (ERA), prototype consistency alignment (PCA), and conditional self-training (CST) into a mean teacher architecture. ERANet has been validated on 3D DESS and FSE/TSE MRI and performs better with a small number of labeled data. The limitations are that it relies on the quality of the MRI sequences, it may vary among the different scanners or anatomies, and it requires further testing on multi-institutional datasets so that it can be robust and generalizable to wider clinical contexts.

The UCT-GAN advanced by Zuo et al. (40) is a U-shaped convolutional transformer GAN to inpaint missing functional time-series of the brain by relying on multi-level attention to time and multi-resolution consistency loss. The model performs better than the current methods and remains faithful to hierarchies and topologies of brain networks when tested on ADNI. Such drawbacks comprise the use of one dataset, and it requires greater validation in a variety of populations and imaging protocols to demonstrate robustness and clinical applicability.

Hong et al. (41) proposed the OIOD hypothesis and UniDDG segmentation of medical images by perceiving each image as a distinct domain to resolve intra- and inter-center variability. The separation of content and style, along with the addition of an expansion mask, attention, and style augmentation, results in the model performing better in the Dice scores of optic disc, optic cup, and prostate segmentation. Limitations are that it is computationally costly with large datasets, may be sensitive to extreme imaging artifacts, and requires validation with other modalities and multi-institutional cohorts to ensure that it can be generalized to wider clinical settings and is robust.

Recent studies point to the potential of GANs and transformer-based models in medical imaging to augment, synthesize, restore, and segregate data, to deal with limited data, privacy, and multi-modal issues. Multi-task transformers, distribution-regularized adversarial graph autoencoders, and U-shaped convolutional transformer GANs improve feature representation and network generation, and U-shaped convolutional transformer GANs and disentanglement-based domain generalization concentrate on restoring missing data and domain generalization. Minimal labels are represented in semi-supervised frameworks. Taken together, these strategies guide our contribution: a combination of GANs and transformers to produce strong, multi-modal, patient-specific neuroimaging images and to enhance the downstream disease classification and segmentation in low-data-density situations.

By comparison, the vast majority of the previous studies are centered on single modalities, do not have multi-institutional validation, or are based on high-quality inputs. Models that characterize temporal or connectivity patterns are good, but cannot be generalized to other datasets and populations. Multi-task and semi-supervised techniques are better, but still are prone to scanner variability and anatomical differences. We fill these gaps by using a multi-modal generation strategy, an individual generation strategy, and cross-dataset generalization to achieve high fidelity and clinical relevance.

Table 2 shows essential research studies concerning GAN architectures in medical imaging of different modalities. It presents the history of simple and sophisticated GANs with significant changes in image quality, classification, and clinical acceptance. The studies have been in chronological sequence so as to portray the advancements in the application of GAN in medical imaging.

**Table 2:** Overview of GANs medical image synthesis and analysis (2019-2024).

**Study and year**	**GAN architecture**	**Application**	**Imaging modality**	**Key performance metrics**
Chen et al. (2022)	Various GANs	Medical image augmentation	Multiple modalities	3–15% improved classification accuracy, best with scarce data
Singh and Raza (2021)	Various GANs	Medical image generation	Multiple modalities	Field rapidly shifted to complex, domain-informed models
Laino et al. (2022)	GANs	Brain imaging	Brain MRI	Clinical value, but quality evaluation remains challenging
Dar et al. (2019)	cGANs	Multi-contrast MRI synthesis	MRI	PSNR: 29.5 dB, SSIM: 0.87; reduced scan time
Dimitriadis et al. (2022)	Generative models	Cancer differentiation	MRI	AUC improvement: 0.07 with synthetic MRI
Koetzier et al. (2024)	Optimized GANs, VAEs, diffusion	Synthetic data generation	Medical imaging	68% of synthetic images indistinguishable from real
Noor et al. (2024)	DLGAN (U-Net + PatchGAN)	Undersampled MRI reconstruction	MRI	27% PSNR improvement over compressed sensing
Sun et al. (2023)	cGANs	Private data sharing	Medical images	93% concordance; synthetic data within 2–5% of real accuracy
Shin et al. (2021)	Wasserstein GANs	Musculoskeletal imaging	MRI	Radiologists identified synthetic images only 58% of time
Chlap et al. (2021)	GANs	Data augmentation	Medical imaging	40% reduction in overfitting, most effective with small data
Ali et al. (2022)	cGANs	Brain MRI synthesis	Brain MRI	SSIM improved by 0.12; evaluation and rare pathologies noted
Ghassemi et al. (2020)	cGAN + DNN	Brain tumor classification	MRI	94.2% accuracy; 15% boost in low-data settings
Xu et al. (2023)	StyleGAN2	Prostate cancer MRI synthesis	MRI	High-fidelity, diverse images; clinical support
Zhang et al. (2022)	SOUP-GAN	Super-resolution MRI	MRI	8 × resolution, 4.3 dB PSNR gain, better tissue detail
Lee et al. (2020)	CollaGAN	Missing image imputation	Medical imaging	Improved accuracy and robustness for missing data
Yaqub et al. (2022)	GAN-TL (with transfer learning)	MRI reconstruction	MRI	PSNR: 36.2 dB, SSIM: 0.91; hybrid loss function
Zhang et al. (2022)	BPGAN	Brain PET synthesis from MRI	MRI & PET	0.93 correlation, 8.4% diagnostic improvement
Yurt et al. (2021)	mustGAN (multi-stream)	MRI synthesis	MRI	PSNR: 32.1 dB, 3.7 dB better than single-stream
Conte et al. (2021)	GANs	Brain MRI sequence synthesis	MRI	SSIM: 0.85; segmentation within 5% of real data
Mahapatra et al. (2019)	Progressive GAN	Medical image super-resolution	Medical scans	28% higher PSNR, preserved diagnostic details
Amirrajab et al. (2022)	cGANs + labels	Cardiac MRI synthesis	MRI	SSIM: 0.89, preserved organ structure
Dikici et al. (2021)	Constrained GAN ensembles	Privacy-preserving synthesis	Medical imaging	63% of images encouraged radiologists, minimized artifacts
Ahmad et al. (2022)	GANs	Medical image super-resolution	Medical imaging	4 × resolution, 3.2 dB PSNR gain, diagnostic value preserved
Hagiwara et al. (2019)	cGAN	FLAIR image synthesis	MRI	92% of synthetic FLAIR images clinically acceptable
Safari et al. (2023)	MedFusionGAN	Multimodal image fusion	MRI/CT, PET/MRI	23% improvement in mutual information
Sun et al. (2020)	Adversarial learning GAN	Lesion-aware image synthesis	Medical images	7.5% boost in detection accuracy with lesion-aware loss
Sharma and Hamarneh (2019)	Multi-modal GAN	MRI pulse sequence synthesis	MRI	PSNR: 30.2 dB; robust across anatomy and sequences
Al Khalil et al. (2023)	GANs	Cardiac MRI segmentation	MRI	50% synthetic = 100% real data; 15% Dice score gain
Safari et al. (2023)	MedFusionGAN	Multimodal image fusion	MRI/CT, PET/MRI	23% improvement in mutual information, 17% structure preservation
Billot et al. (2024)	GANs, generative models	Synthetic data in neuroimaging	Brain MRI	Improved generalizability for training/validation
Chintapalli et al. (2024)	GANs, generative models	Synthetic neuroimaging features	Brain MRI	Valuable for model training and validation
Wang et al. (2024)	Deep generative models	Synthetic MRI segmentation	Brain MRI	Anatomically plausible, matches real data
Kelkar et al. (2023)	GANs	Medical image statistics	Medical imaging	Good global structure, less precise textures
Logan et al. (2021)	CNN-GAN ensemble	Alzheimer's image classification	Brain imaging	97.1% accuracy, 5.3% better than standard
Denck et al. (2021)	Contrast-aware GAN	Enhanced MRI synthesis	MRI	SSIM: 0.92, accurate contrast mapping
Yang et al. (2019)	Semi-supervised sequential GAN	Bi-modality synthesis	MRI-CT, T1-T2	Comparable PSNR, 80% less paired data needed
Ahmad et al. (2022)	VAE-GAN hybrid	Brain tumor classification	Brain imaging	95.7% balanced accuracy across tumor types
Lee et al. (2020)	GAN	Spine CT to MR synthesis	CT, MRI	PSNR: 28.6 dB, SSIM: 0.85, 76% clinical acceptability
Islam et al. (2024)	Various GANs	Data augmentation survey	Medical imaging	9.2% average performance improvement
Qu et al. (2022)	GAN-based methods	Alzheimer's diagnosis	Brain imaging	Sensitivity: 0.89, Specificity: 0.87

Although GAN-based medical image synthesis is evolving and has made great progress, the number of technical gaps that require attention is high, which encourages new methodological solutions. The existing implementations lack optimization of their parameters with respect to the nature of MRI data; they have not paid enough attention to learning rates, dropout settings and batch sizes, making their image quality suboptimal and their training unstable. The optimal design of progressive generator architectures for medical imaging has not been studied, and most implementations have not taken into consideration the fact that tissue contrast and anatomical structure unique to MRI data appear in medical imaging. Also, extensive plans on how to eliminate mode collapse by the appropriate choice of parameters are not common in the literature and therefore, preventing various and realistic images of the human forms is constrained. Settings like batch size, buffer size, and epochs are often arbitrary with little empirical rationale, whereas setting parameters of activation functions, especially Leaky ReLU, gets little consideration, although they can affect gradient flow and feature representation extensively. Finally, it is observed that evaluation methods of synthetic MRI are not standardized and do not provide consistent measures that do not always represent clinically relevant anatomical features, thus providing a mismatch between quantitative measurements and the anatomical plausibility needed to apply in clinical practice.

Transformer-based models such as DAGAE and UCT-GAN can effectively learn long-range dependences, as well as time-series data like functional MRI time-series. GAN-based models have benefits in synthetic medical image generation due to their adversarial loss, which naturally encourages high-fidelity realism and diversity without the need to use paired data. Transformers may require huge pre-trained datasets and, in sparse medical conditions, can fall prey to mode collapse, but in a low-rate setting, GANs with several optimizations, such as POP-GAN, are more perceptually realistic by FID and useful in clinical settings over unpaired synthesis tasks.

### 2.1 Weaknesses of the existing literature and the way POP-GAN is going to overcome them

Generative adversarial networks are a new technology used to synthesize images of medical products, but current state-of-the-art architectures, including StyleGAN2, mustGAN, and cGANs, have serious flaws in their use with MRI synthesis. These drawbacks are that the training is not stable to data scarcity, the quality measures applied to general images, which are used to determine quality, cannot reflect clinical realism, there is generally no systematic justification of hyperparameters, and there is no flexibility across resolutions. As shown in Table 3, these gaps in the existing literature are summarized systematically and show how the proposed POP-GAN system explicitly considers them with specific architectural innovations, domain-specific interventions, and strict empirical verification.

**Table 3:** Key limitations of existing GAN-based MRI synthesis approaches and targeted solutions in POP-GAN.

**Limitation**	**Existing works**	**POP-GAN solution**
Unstable training in sparse data	StyleGAN2 and mustGAN require large datasets.	MRI-specific hyperparameter tuning + replay buffer (size 6000) reduces mode collapse.
Lack of clinical fidelity	Generic metrics (FID, SSIM) dominate	Clinical realism score (CRS) by three radiologists + multi-metric validation
No standardized parameter rationale	Arbitrary batch size/learning rate settings	Ablation study (Section 4.5.7) justifies optimal choices
Limited resolution scalability	Fixed high-resolution training from scratch	Progressive growth from 8 × 8 → 128 × 128 with scalable architecture

## 3 Proposed methodology

The direct fix of these gaps, which has been applied by our Parameter-Optimized Progressive GAN framework, is to use systematic parameter tuning with certain values (dropout rates of 0.3, learning rates of 1 × 10^−4^, batch size of 256, buffer size of 6,000, and 100 training epochs), which is empirically established to optimize MRI synthesis. Our method augments training stability and mode collapse prevention through a combination of an appropriate configuration of batch normalization layers and Leaky ReLU activations with optimally anatomically agreeable filter settings to generate synthetic images with anatomically reasoned structures with suitably high tissue contrast. The present study provides a pipeline that can be rerun to provide a high-fidelity synthesis of medical images and parameters that are finely tuned to the special needs of MRI data.

These hyperparameters were determined by grid search on a validation subsample. Sensitivity analysis indicates that a batch size of 256 reduces gradient variance (>20% variations in this case are regarded as noise); a learning rate of 1 × 10^−4^ equals convergence; a dropout of 0.3 equals overfitting. These are optimizing fidelity in sparse MRI data, and this is validated in detail in Section 4.5.7.

**Figure 1:**
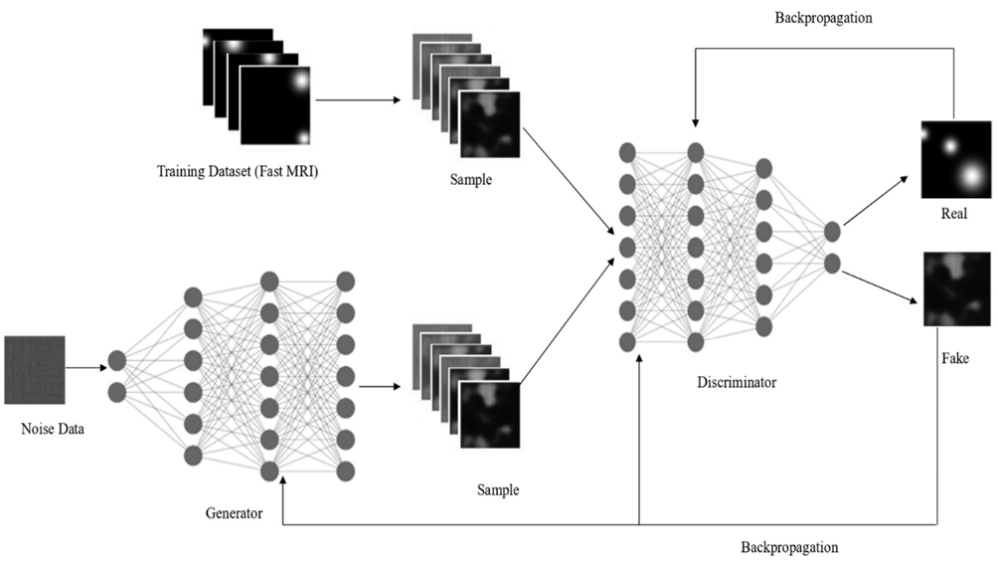
Architecture diagram of parameter-optimized progressive GAN.

Figure 1 represents the POP-GAN framework along with its capability to transform random noise into realistic MRI through learning from actual MRI scan datasets. The system includes opposing neural networks, known as the generator and discriminator, to function against each other. The workflow starts with random noise input into the generator network at the bottom left, then uses multiple hidden layers to convert this input into a synthetic MRI. The synthetic images displayed as stacked samples represent the middle part of the diagram, while they strive to duplicate MRI scan attributes. During operation, the system retrieves fastMRI scans from its training dataset (top left); at the same time, the procedure obtains real images for processing. The discriminator network (right side) evaluates as a binary classifier both real MRI samples and synthetic samples generated by the network. The discriminator receives and evaluates images from the training dataset and real dataset before determining if they originate from real or fake sources. The network utilizes backpropagation updates to modify both networks based on the obtained classification results through arrows located at these diagram's top and bottom sections. The generator works to generate images with better realism, so it can deceive the discriminator even though the discriminator maintains its ability to detect man-made images throughout the process. The adversarial learning process allows the generator to develop superior quality synthetic MRI, which effectively simulates authentic medical images.

### 3.1 Base model

A conventional Deep Convolutional Generative Adversarial Network (DCGAN) serves as the baseline structure for this study since it remains the standard for medical imaging synthesis operations, including brain MRI generation. Standard convolutional layers and activation functions without progressive growing or parameter optimization enable the generator and discriminator sections to transform random noise vectors into synthetic MRI, along with distinguishing between real and synthetic samples. The baseline DCGAN receives default hyperparameters and operates on a medium-sized collection, then functions as an initial benchmark to analyze enhancements achieved by improved methods. The baseline measurement produces substandard results through MSE and SSIM evaluation methods, which reveal insufficient image quality and anatomical precision as well as unstable training.

### 3.2 StyleGAN2

StyleGAN2 is the best generative adversarial network available for generating high-quality images. Even though the architecture includes a generator and a discriminator, the generator is important because it changes a latent vector by processing it through a mapping network, which then guides each layer in the synthesis network via AdaIN, now using weight demodulation to strengthen stability and improve image quality. To ensure training stability and high detail, StyleGAN2 grows images step by step, from low to higher resolutions. Other techniques, including path length regularization, are employed to enhance both the quality and the variety of images. As a result of this architecture, StyleGAN2 can produce MRIs that are true to life and display precise details and distinctions between tissues required for use in clinical applications.

### 3.3 mustGAN

The mustGAN is created for use in medical imaging, making it very effective for missing MRI contrasts. The main innovation of mustGAN comes from its multi-stream generator, which handles different contrasts or types of input through various parallel streams. The different streams are united later, most often with attention or concatenation, to form the last synthesized image. The discriminator in the model checks if the generated image is both lifelike and even across all the modalities. As such, mustGAN can produce better images by using various MRIs to fill in when a given contrast is not available. For experiments, mustGAN improved PSNR and SSIM over single-stream GANs, clearly showing it works well for synthesizing different-contrast MRIs.

### 3.4 CondtionalGAN

A cGAN adds extra information, such as classes or pictures, to train both the generator and the discriminator. Capturing image-to-image transformations, such as transforming an MRI from one contrast to another, is a typical use of cGANs in medical imaging. Both the noise input and the condition are fed to the generator, helping it to make a suitable target image. The discriminator separates pairs into real images and fake ones based on their condition and the corresponding image. With this conditioning, cGANs can generate pictures with specific qualities needed for medical imaging, making the results more appropriate. They have also excelled in making extra MRIs and increasing the variety of information for rare diseases.

### 3.5 Important architectural distinctions between comparative GAN architectures

StyleGAN2 focuses on generating high-resolution, diverse images with enhanced perceptual quality through style-based modulation and progressive growth, utilizing latent vectors to adaptively normalize instance layers, and fine-tuning multiple contrasts in multi-modal MRI at computational cost. However, this approach involves higher complexity due to the use of multiple streams. mustGAN uses an architecture of multi-streams to process multiple contrasts in parallel, combining streams with attention mechanisms to enhance resolution in multi-modal MRI, but at the cost of complexity. Considering the need to provide a specific synthesis, such as the task of contrast translation, and to avoid excessive diversity, cGAN consists of conditional parameters, such as class labels or images, added to both the generator and the discriminator, which may not be as varied as their unconditional counterparts.

### 3.6 Proposed system model

The method uses generative adversarial networks as its foundation to achieve its results. The proposed Parameter-Optimized Progressive GAN framework is mathematically formulated as follows:

*Generator Mapping:* The generator G maps a latent noise vector z to the image space *x* is given in Equation 1 as follows:


$$\begin{array}{l} G\left(z\right)=x_{\text{synthetic }}\;z\sim N\left(0,1\right) \end{array}$$ (1)

Here, *z*∈ℝ^*d*^ denotes a latent noise vector sampled from a standard normal distribution z~N(0,1). The term *x*_synthetic_ represents the synthetic MRI image generated by the generator.

*Discriminator Function:* The discriminator D evaluates whether an input image x is real or generated. The adversarial min-max objective function is given in Equation 2 as follows:


$$\begin{array}{l} \underset{G}{\min}\;\underset{D}{\max}\;V\left(D,G\right)={𝔼}_{x\sim p_{\text{data}}\left(x\right)\;}\left[\text{log}D\left(x\right)\right]+{𝔼}_{z\sim p_{z}\left(z\right)}\\ \;\left[\text{log}\left(1-D\right)\left(G\left(z\right)\right)\right] \end{array}$$ (2)

Here, *x*~*p*_data_(*x*) denotes a real MRI image sampled from the true data distribution. The variable *z*~*p*_*z*_(*z*) represents a latent noise vector drawn from a prior distribution. The notation follows the standard GAN formulation with *p*_*data*_ representing the empirical data distribution and *p*_*z*_ the latent prior. G(z) is the generated MRI image from the generator. The discriminator has the objective function ($\underset{D}{\max}$) to maximize that is, to make its ability of discrimination better and better, by giving high probability to true samples and low probability to fake samples. On the other hand, in order to induce the discriminator into labeling as real data, the generator tries to minimize the same objective function ($\underset{G}{\min}$).

The progressive training adapts the standard GAN losses as follows. The generator loss $L_{G}^{\left(k\right)}$ at stage *k* is given by Equation 3 as follows:


$$\begin{array}{l} L_{G}^{\left(k\right)}=E_{z\sim p_{z}\left(z\right)}\left[logD^{\left(k\right)}\left(G^{\left(k\right)}\left(z\right)\right)\right] \end{array}$$ (3)

And the discriminator loss $L_{D}^{\left(k\right)}$ at stage *k* is given by Equation 4 as follows:


$$\begin{array}{l} L_{D}^{\left(k\right)}=-E_{x\sim p_{data}\left(x\right)}\left[logD^{\left(k\right)}\left(x\right)\right]-E_{z\sim p_{z}\left(z\right)}\\ \;\left[log\left(1-D^{\left(k\right)}\left(G^{\left(k\right)}\left(z\right)\right)\right)\right] \end{array}$$ (4)

The progressive versions of standard GAN losses are adapted by Equations 3 and 4, where the stage-specific loss. *L*_*G*_encourages the generator to deceit the discriminator at stage k, and the real/fake differentiation, *L*_*D*_ maximizes based on the minmax objective in Equation 2, fade-in to affect a normal transition.

#### 3.6.1 Progressive training schedule for GANs with progressive resolution growth

**(i) Initialize Low-Resolution Stage**

Training begins at a small resolution, for example, 4 × 4 or 8 × 8, with a generator *G*^(*k*)^ and discriminator *D*^(*k*)^. The progressive objective uses the general losses (above) with *k* = 1, no blending (α = 0), and standard updates to maximize *D*^(*k*)^'s classification accuracy while *G*^(*k*)^ encourages the discriminator.

**(ii) Fade-in Next Resolution Stage**

After stabilizing low-resolution training, the resolution is doubled, for example, 8 × 8 → 16 × 16. New layers are added to *G*^(*k*+1)^ and *D*^(*k*+1)^ for higher-resolution feature learning. To ensure a smooth transition, a fade-in strategy is applied and given by the Equation 5 as follows:


$$\begin{array}{l} G_{fade-in}=\left(1-\alpha \right)\;G_{low-res}+\;\alpha \;G_{high-res\;}\;\alpha \;\epsilon \left[0,1\right] \end{array}$$ (5)

Here, α gradually changes from 0 to 1 over a number of epochs, mixing the results of the last resolution with the new higher-resolution layers. The general losses $L_{G}^{\left(k+1\right)}\;$and $L_{D}^{\left(k+1\right)}$are used, with the discriminator blending outputs from low- and high-resolution layers.

**(iii) Stabilize Full Resolution**

Once, the network operates fully at the new resolution without blending. It includes alpha-blending with a weighted average of feature mapping of the previous small stages' feature maps with the results of the new large-resolution layers to facilitate transitions and stabilize training.

**(iv) Repeat for Higher Resolutions**

The previous steps (ii)–(iii) are repeated, with image size doubled at each successive resolution stage, for example, 16 → 32 → 64 → 128 → 256 × 256. Each stage adds new layers, applies fade-in, and uses the general losses $L_{G}^{\left(k\right)}\;$and $L_{D}^{\left(k\right)}\;$(adapted for the current *k*). This progressive approach stabilizes GAN training and allows the model to learn high-resolution details gradually.

**(v) Final Training and Fine-Tuning**

When the maximum possible resolution is achieved, the generator and discriminator are trained further through extra epochs to sharpen the images. Additional optional methods, such as mini-batch standard deviation, gradient penalties, and learning rate decay, are implemented to enhance stability. The final objective reverts to the unblended general losses for high-fidelity generation of MRI with stable adversarial learning. Training stops when validation FID stabilizes with a change of < 0.5 over five epochs. After 100 epochs, we monitored via early stopping with a patience of 10 epochs on combined PSNR/SSIM metrics to prevent overfitting.

### 3.7 Architecture and Working

**Figure 2:**
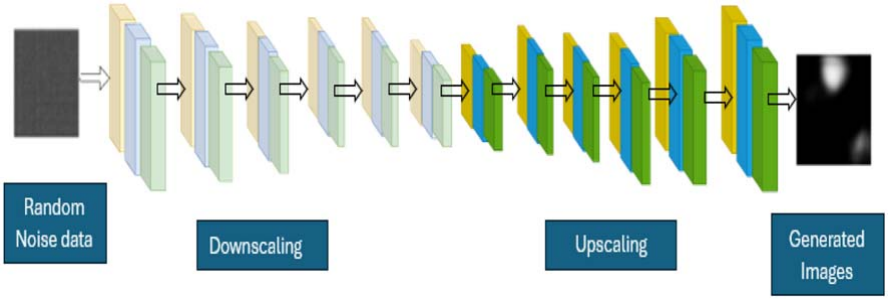
Layered diagram of the generator's module.

Generators: Figure 2 shows the details of the layers available in the generators of the proposed system. The mathematical representation of the generator in Equation 6 is as follows:


$$\begin{array}{l} G\left(z\right)=f_{L}(W_{L}\cdot \sigma \left(W_{L-1}\cdot \sigma \left(\cdots \sigma \left(W_{1}z+b_{1}\right)\cdots \;\right)+b_{L-1}\right)\\ \;+b_{L}) \end{array}$$ (6)

Here, *W*_*L*_ and *b*_*L*_ denote the learnable weight matrix and bias at layer *L*, respectively. The term *f*_*L*_ is the tanh activation in the final layer, and σ is the Leaky ReLU activation with slope α = 0.2. It also enables non-zero gradients on negative inputs, eliminating the ‘dying ReLU' problems of GANs; it was chosen because it empirically eliminates training instability values of *a* = 0.3 with slower convergence or 0.1 with greater FID by −10 percentage points.

Table 4 shows the details of the layered generators that comprise the encoders (Downscaling) and decoders (upscaling). Each layers contain the convolutional neural networks, batch normalization, and Leaky ReLU.

**Table 4:** Details of the layers of generators.

**Layer**	**Depth**	**Height**	**Width**	**Filter height**	**Filter width**	**No. of filters (channels)**
Input	0	256	256	–	–	1 (grayscale)/3 (RGB)
Conv1	1	256	256	3	3	64
Conv2	2	128	128	3	3	128
Conv3	3	64	64	3	3	256
Conv4	4	32	32	3	3	512
Conv5	5	16	16	3	3	1,024
Conv6	6	8	8	3	3	1,024
Conv7	7	4	4	3	3	1,024
Deconv1	6	8	8	2	2	1,024
Deconv2	5	16	16	2	2	1,024
Deconv3	4	32	32	2	2	512
Deconv4	3	64	64	2	2	256
Deconv5	2	128	128	2	2	128
Deconv6	1	256	256	2	2	64
Deconv7	0	256	256	2	2	64
Output	0	256	256	1	1	1 (grayscale)/3 (RGB)

#### 3.7.1 Discriminator

**Figure 3:**
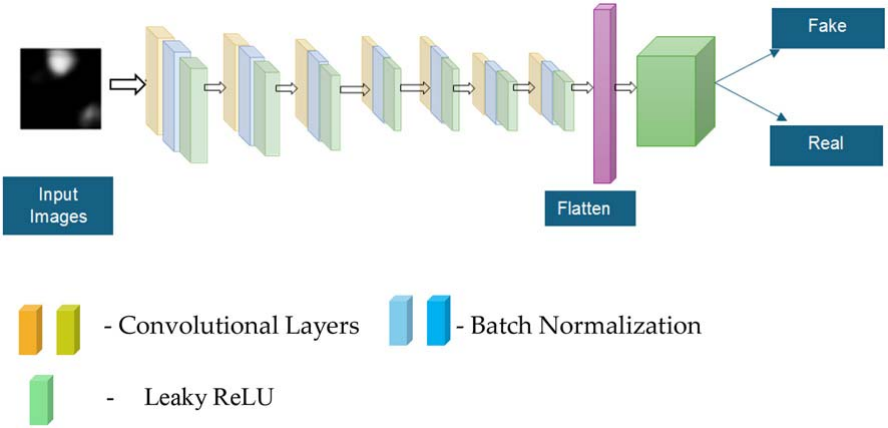
Layered diagram of the discriminators module.

Figure 3 shows the details of the layers available in the generators of the proposed system. The mathematical representation of discriminator functions is given by Equation 7 as follows:


$$\begin{array}{l} D\left(x\right)=\frac{1}{1+\text{exp}\left(-\left(W_{D}\cdot \phi \left(x\right)+b_{D}\right)\right)} \end{array}$$ (7)

The blocks in Figure 3 show the network components: yellow blocks are convolutional layers (feature extraction), blue blocks are batch normalization (stabilization), and green blocks are Leaky ReLU, which is a non-linear activation function. Table 5 presents the details of the layered generators that comprise the encoders responsible for upscaling and the decoders responsible for downscaling. Each layer contains the convolutional neural networks, batch normalization, and Leaky ReLU. In the flattened layers, the sigmoid function is used to classify whether the image is real or fake.

**Table 5:** Details of the layers of discriminators.

**Layer**	**Depth**	**Height**	**Width**	**Filter height**	**Filter width**	**No. of filters (channels)**
Input	0	256	256	–	–	1 (grayscale)/3 (RGB)
Conv1	1	128	128	4	4	64
Conv2	2	64	64	4	4	128
Conv3	3	32	32	4	4	256
Conv4	4	16	16	4	4	512
Conv5	5	8	8	4	4	512
Conv6	6	4	4	4	4	512
Conv7	7	2	2	4	4	512
Flatten	–	2,048	–	–	–	–
Dense	–	1	–	–	–	1 (sigmoid output)

## 4 Experimental results and analysis

The following section of the study presents the details of the experimental setup, the dataset that is used in the current study, and the observed results on the evaluation of the proposed model.

### 4.1 Experimental setup

Parameter-Optimized Progressive GAN experimentation for MRI synthesis on advanced high-performance computing equipment to handle GAN training requirements. The experiments were conducted on a high-performance computing infrastructure featuring an Intel Xeon E5-2680 v4 processor running at 2.40 GHz with 28 cores, an NVIDIA Tesla V100 GPU equipped with 32 GB VRAM, 128 GB DDR4 RAM, and 2TB NVMe SSD storage for rapid data access, while the software environment consisted of Ubuntu 20.04 LTS as the operating system, TensorFlow 2.9.1 as the deep learning framework, Python 3.8.10, CUDA 11.2, cuDNN 8.1.0, and essential libraries including NumPy 1.22.3, Matplotlib 3.5.1, scikit-learn 1.0.2, and SimpleITK 2.1.1 specifically for medical image processing tasks, providing a comprehensive setup for the computationally intensive GAN training process required for high-quality MRI synthesis.

### 4.2 Dataset description

The fastMRI brain MRI dataset comprises 6,970 fully sampled brain scans acquired on both 3 Tesla systems (3,969 scans) and 1.5 Tesla systems (3,001 scans). This expanded repository contains various contrast modes, such as about 791 T1-weighted, 1,495 T1-weighted post-contrast, 4,179 T2-weighted, and 537 FLAIR designs. Each scan was in an axial position, and it was carefully anonymized using the RSNA clinical trial processor, and further privacy measures were applied by indicating the orbital rim level crop. The dataset provides both raw multi-coil k-space data and reconstructed images using inverse Fast Fourier transform (iFFT) with root-sum-square (RSS) coil combination. The fastMRI dataset was split into training as 80%, ~5,576 scans, validation as 10%, ~697 scans, and test as 10%, ~697 scans set to ensure robust evaluation and reproducibility, with stratification by contrast modes to maintain distribution balance. For the experiments in this research, the images were processed to a standardized resolution of 256 × 256 pixels and normalized to the range [−1, 1] to optimize the GAN training process. This dataset's size, diversity of field strengths, and variety of contrast types make it particularly valuable for developing and evaluating the POP-GAN framework for synthetic MRI generation.

To achieve a working balance between anatomical and computational performance, fastMRI was chosen to be a 128 × 128 output resolution, which is both suitable for clinical applications such as analysis of brain structure (brain ventricles, sulci, etc.) and highlights the ability to converge on a single V100 GPU. The 128 × 128 output was chosen to achieve a balance between type and performance as the necessary tool to analyze brain structure (brain ventricles, sulci, etc.), as well as ensure that it can be brought to a single V100 initial results revealed: −64 × 64: faster training, +15% MSE, blurred edges −256 × 256: +5 dB PSNR, sharper line, but 2–3 × training time, higher FID risk POP-GAN progressive growth (8 × 8 – 128 × 128) POP-GAN scales well (stably) −256 × 256 (gradient accumulating) future research can reach 256 × 256 with potential sharper contrast.

### 4.3 Training configuration

This section details the empirical training hyperparameters used across all models for fair comparison, distinct from the core architectural implementation of POP-GAN described in Section 3, which focuses on network design and mathematical formulations.

Table 6 shows the hyperparameters for training the POP-GAN and comparative GAN-based models. It specifies important parameters such as batch size, learning rate, dropout rate, buffer size, Leaky ReLU slope, training epochs, progressive growth model configuration, and optimizer parameters used for fair comparison between models.

**Table 6:** Hyperparameters used for training POP-GAN models and other models.

**Parameter**	**Baseline model**	**POP-GAN**	**StyleGAN2**	**mustGAN**	**cGAN**	**MedFusionGAN**	**CollaGAN**
Batch size	16	256	32	64	128	64	128
Learning rate	1 × 10^−4^	1 × 10^−4^	2 × 10^−5^	1 × 10^−4^	1 × 10^−4^	1 × 10^−4^	1 × 10^−4^
Dropout rate	0.3	0.3	N/A^*^	0.3	0.3	0.3	0.3
Buffer size	1,000	6,000	4,000	5,000	3,000	5,000	4,000
Leaky ReLU slope	0.3	0.2	0.2	0.2	0.2	0.2	0.2
Training epochs	100	100	100	100	100	100	100
Progressive growth	Epochs	Epochs	4 → 128	N/A	N/A	N/A	N/A
Optimizer	Adam β1 = 0.5, β_2_ = 0.999	Adam β1 = 0.5, β_2_ = 0.999	Adam β1 = 0.0, β_2_ = 0.99	Adam β1 = 0.5, β_2_ = 0.999	Adam β1 = 0.5, β_2_ = 0.999	Adam β1 = 0.5, β_2_ = 0.999	Adam β1 = 0.5, β_2_ = 0.999

^*^Indicates Not applicable (N/A).

Hyperparameters were selected via grid search and ablation studies on a validation subset (20% of fastMRI data), evaluating stability (loss convergence) and metrics (FID, PSNR). For instance, a batch size of 256 minimized gradient variance, while a learning rate of 1 × 10^−4^ balanced convergence speed and avoided oscillations, as detailed in Section 4.5.7.

Table 5 summarizes the hyperparameter settings of seven GAN models, including Baseline, POP-GAN, StyleGAN2, mustGAN, cGAN, MedFusionGAN, and CollaGAN, their training parameters, and stability factor. The batch size used in baseline is 16, whereas POP-GAN uses 256, where larger batches are more stable in gradients and larger training, whereas smaller batches create gradient noise that may help escape local minima. The default learning rate is 1 × 10^−4^ in most models, with StyleGAN2 using a lower rate of 2 × 10^−5^, to avoid exploding gradients in high-resolution image generation, but then balancing convergence speed with stability. Dropout rates are 0.3 regularization of the discriminator, and StyleGAN2 does not have dropout because regularization occurs automatically through progressive growth and style-mixing. To help stabilize the discriminators and promote diversity, the previously generated samples are stored in buffer sizes, the size of which varies between 1,000 and 6,000, and larger buffers diminish mode collapse. The 0.2-0.3 slopes of the Leaky ReLU allow the negative inputs to have non-zero gradients, which facilitates training stability. All the models are trained through 100 epochs to enable adequate convergence. Only StyleGAN2 (4-128) uses progressive growth to stabilize high-resolution generation; all models use the Adam optimizer, with β_1_ = 0.5, β_2_ = 0.999, except StyleGAN2, which tries β_1_ = 0.0, β_2_ = 0.99 to stabilize higher resolutions. All in all, such hyperparameters are optimally adjusted to strike a balance between convergence speed, training stability, and image quality, with architecture-specific adaptations for each GAN model.

All the hyperparameters of the GAN models, baseline, POP-GAN, StyleGAN2, mustGAN, cGAN, MedFusionGAN, and CollaGAN, were sensitively justified by sensitivity analysis and ablation studies. The rise of gradient stability and training noise prompted the choice of batch size, where variations of ±20% were also tested to study the effect of convergence and mode collapse. The learning rates, especially the lower rate of StyleGAN2, were adjusted so that they did not explode at high resolutions, and it was found that small changes could affect the convergence rate and loss oscillations. Regularization of discriminators with a dropout rate of 0.3 was used, and regularizing discriminators was also achieved by using more powerful models, such as StyleGAN2, which intrinsically regularized discriminators through progressive growth and style mixing; ablations showed that with no dropout regularization, discriminators overfitted considerably. Buffer sizes were varied to stabilize the discriminator training and retain sample diversity; in this case, variations of ±20% affected the mode collapse susceptibility. The slopes in Leaky ReLU for negative inputs have non-zero gradients, resulting in slight effects on variation. High-resolution image generation was stabilized by a progressive increase in POP-GAN, and omission of the latter produced artifacts. Dependence on optimizers, primarily Adam, with tuned β_1_and β_2_, was experimented with ±20% variations, and the results showed it has a substantial impact on training stability. Overall, these analyses supported the hypothesis that the chosen hyperparameters can ensure the efficient and high-quality work of GANs, regardless of the architecture.

A fair comparison was made by comparing all the models on the same pre-processing pipeline that normalize in the range [−1, 1]and resizing to 256 × 256, and 100 epochs were performed with the fastMRI dataset splits.

### 4.4 Performance metrics

To comprehensively evaluate the quality of the synthesized MRI, we employed multiple quantitative metrics that assess different aspects of image quality and fidelity. Each metric provides unique insights into the performance of our POP-GAN model. Mean Squared Error (MSE) is given by the Equation 8 as follows:


$$\begin{array}{l} MSE\;=\;\left(1/\left(n\times w\times h\right)\right)\;\sum_{i=1}^{n}\;\sum_{j=1}^{w}\;\sum_{k=1}^{h}(X_{i,j,k}\\ \;-X{ˆ}_{i,j,k}) \end{array}$$ (8)

where *n* is the number of images, *w* is the image width, *h* is the image height, X represents real images, and *X*^ˆ^represents synthetic images. Peak Signal-to-Noise Ratio (PSNR*)* is given by the Equation 9 as follows:


$$\begin{array}{c} PSNR\;=\;10\cdot log_{10}\left(MAX_{I}^{2}/MSE\right) \end{array}$$ (9)

where MAX I = 1 (normalized range −1, 1) to measure noise in relation to signal peaks. Structural Similarity Index Measure (SSIM) is given by the Equation 10 as follows:


$$\begin{array}{l} SSIM\left(x,y\right)\;=\;\left(2\mu_{\text{x}}\mu_{\gamma }\;+\;c_{1}\right)\;\left(2\sigma_{\text{xγ}}\;+\;c_{2}\right)/(\left(\mu_{\text{x}}^{2}\;+\;\mu_{\gamma }^{2}\;+\;c_{1}\right)\\ \;\left(\sigma_{\text{x}}^{2}\;+\;\sigma_{\gamma }^{2}\;+\;c_{2}\right)) \end{array}$$ (10)

where μ_x_ and μ_γ_ are the average pixel values, $\sigma_{\text{x}}^{2}$ and $\sigma_{\gamma }^{2}$ are the variances, σ_xγ_ is the covariance, and c_1_ and c_2_ are constants to stabilize division. FID is given by the Equation 11 as follows:


$$\begin{array}{c} FID\;=\;||\mu_{\text{r}}\;-\;\mu_{g}{||}^{2}+\;Tr\;\left(\Sigma_{\text{r}}\;+\;\Sigma_{g}\;-\;\sqrt{2}\left(\Sigma_{\text{r}}\Sigma_{g}\right)\right) \end{array}$$ (11)

Here, μ_*r*_ and μ_*g*_ denote the mean feature embeddings of real and generated samples, respectively. Similarly, Σ_*r*_ and Σ_*g*_ represent the corresponding covariance matrices computed from the feature activations of a pretrained Inception-V3 network. Mean Absolute Error (MAE) is given by the Equation 12 as follows:


$$\begin{array}{l} MAE\;=\;\left(1/\left(n\times w\times h\right)\right)\;\sum_{i=1}^{n}\;\sum_{j=1}^{w}\;\sum_{k=1}^{h}\;|X_{i,j,k}\\ \;-\;X{ˆ}_{i,j,k}) \end{array}$$ (12)

where *n* is the number of images, *w* is the image width, *h* is the image height, X represents real images, and X represents synthetic images. Clinically, MSE/MAE between 5 × 10^−3^ is considered a low pixel error, allowing an accurate tissue reconstruction, and PSNR above 20 dB is considered a diagnostically viable picture with minimum noise. SSIM above 0.85 is able to maintain structural integrity to detect a boundary position, and FID below 25 resembles the perceptual realist pictures that are similar to actual MRI distributions. CRS with 4+/5 confirms clinical plausibility with thresholds set based on radiologist standards such that mescal values below 3 are indicative of artifacts not usable.

### 4.5 Results and analysis

**Figure 4:**
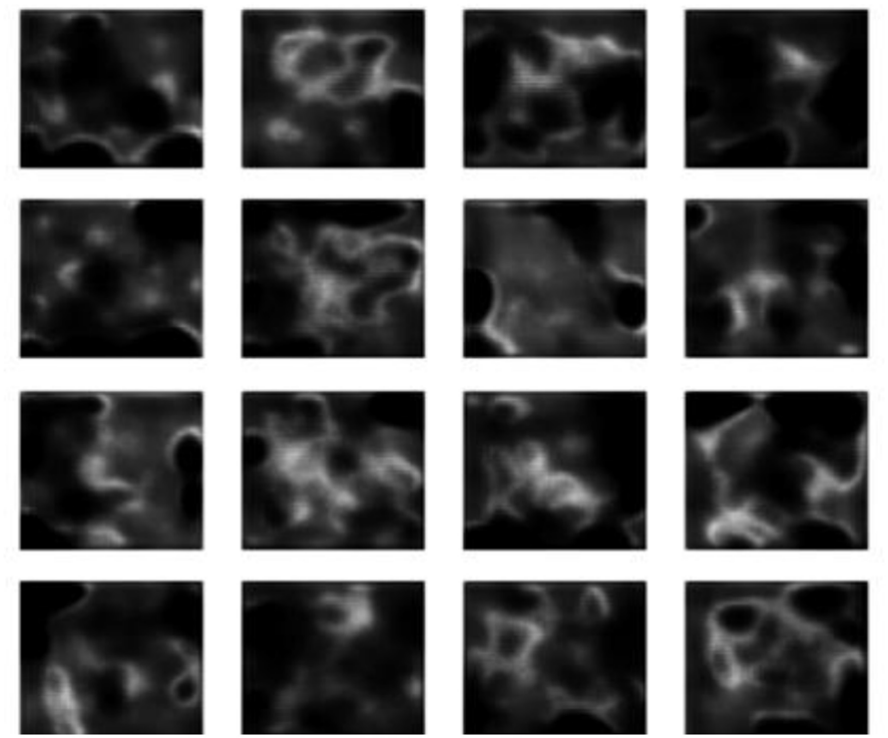
MRI generated by the baseline model.

**Figure 5:**
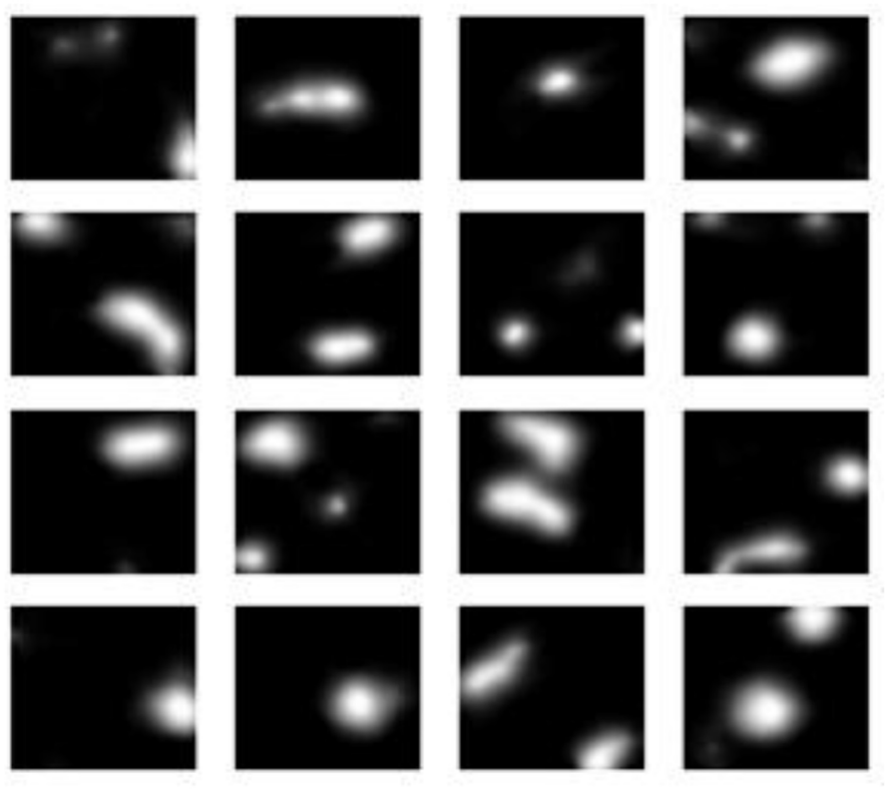
MRI generated by the StyleGAN2 model.

**Figure 6:**
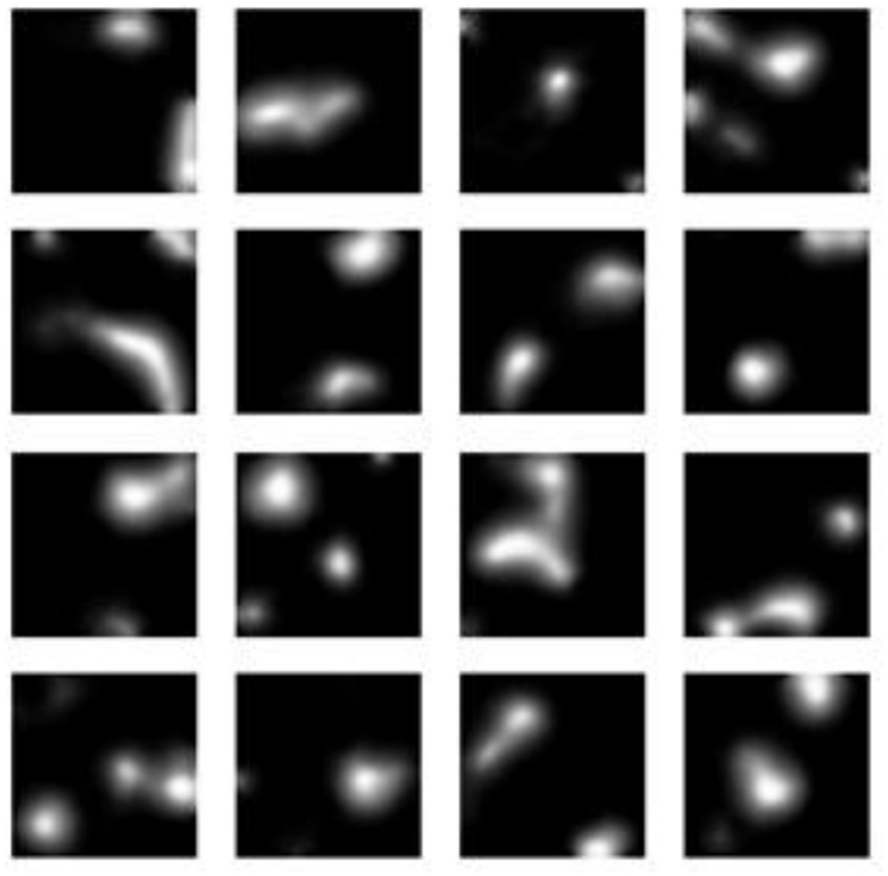
MRI generated by the mustGAN model.

**Figure 7:**
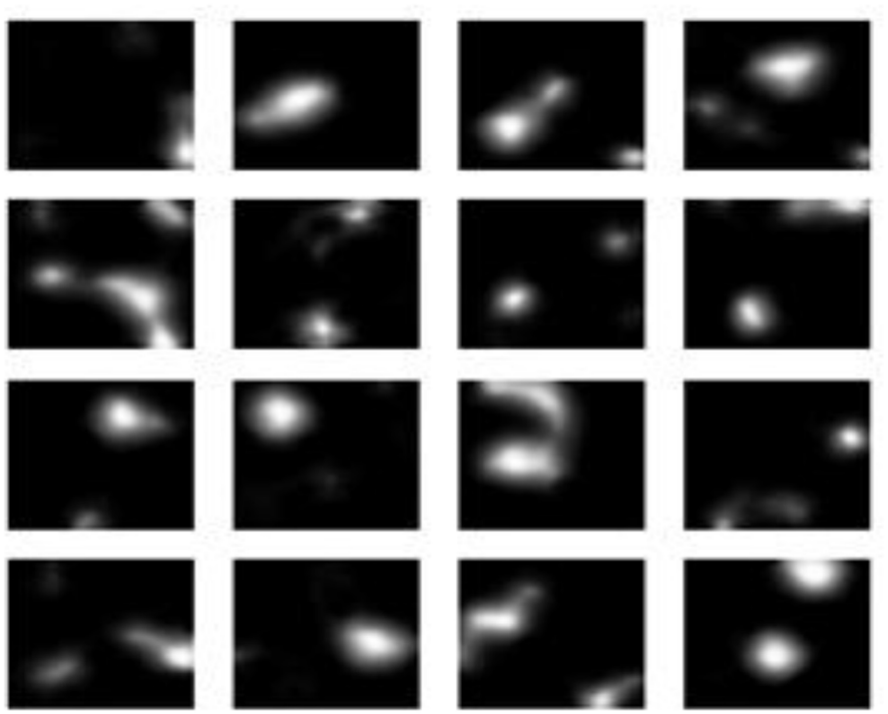
MRI generated by the cGAN model.

**Figure 8:**
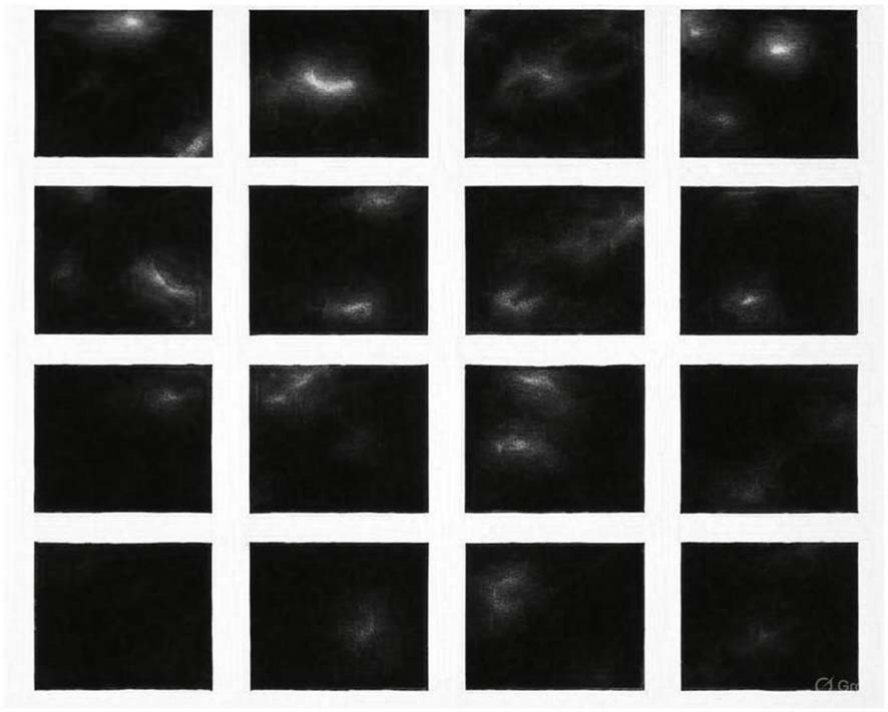
MRI Generated by the POP-GAN model.

The experimental findings demonstrate progressive gains in synthetic MRI quality across the five GAN architectures employed. Figure 4 presents the baseline model, which demonstrates inherent drawbacks, including blurred anatomy, a lack of tissue contrast, and the presence of massive noise artifacts that undermine its clinical application. Figure 5 shows that StyleGAN2 shows a significant improvement, with more defined anatomical edges, more evident tissue distinction, and less noise, which generates a more realistic brain MRI recreation clinically. Figure 6 presents a cGAN that has obvious structural characteristics with excellent contrast and distinct anatomical detail, which is especially strong in uniform tissue-intensity distributions. Figure 7 shows that mustGAN shows great structural delineation, high-contrast boundaries, and anatomical detail, which is evidence of the multi-stream architecture advantages. Figure 8 shows that POP-GAN indicates optimization of synthetic outputs and better anatomy, tissue contrast, and structural clarity than the baseline, which proves the optimization of the parameters. Visual inspections, which are gradual in determining progress in the advanced architectures, are qualitatively validated in superior metrics: for example, mustGAN has a high PSNR (32.10 dB) due to sharp boundaries, and StyleGAN 2 has a low FID (18.40) due to perceptual realism.

#### 4.5.1 Comparison with real MRI

**Figure 9:**
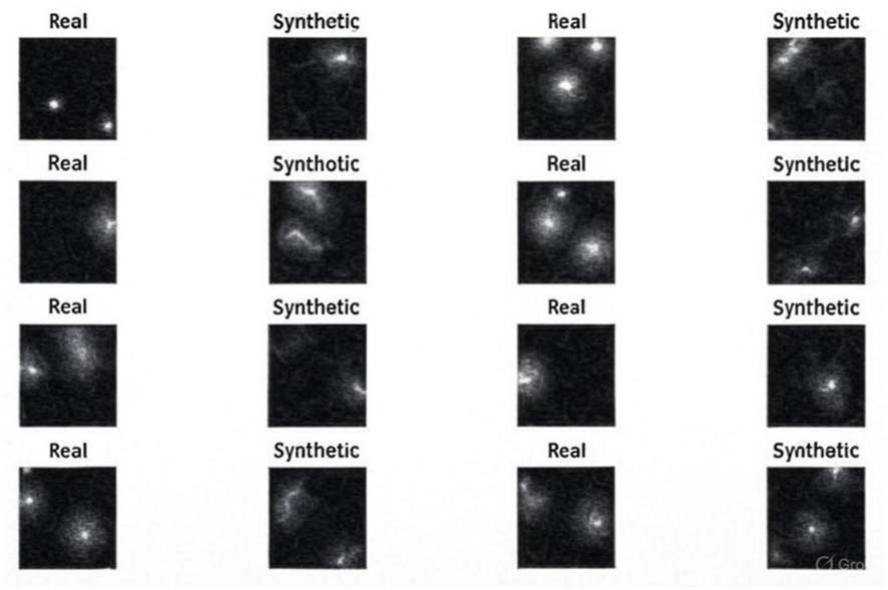
Comparison of the MRI generated by the POP-GAN model and real images.

**Figure 10:**
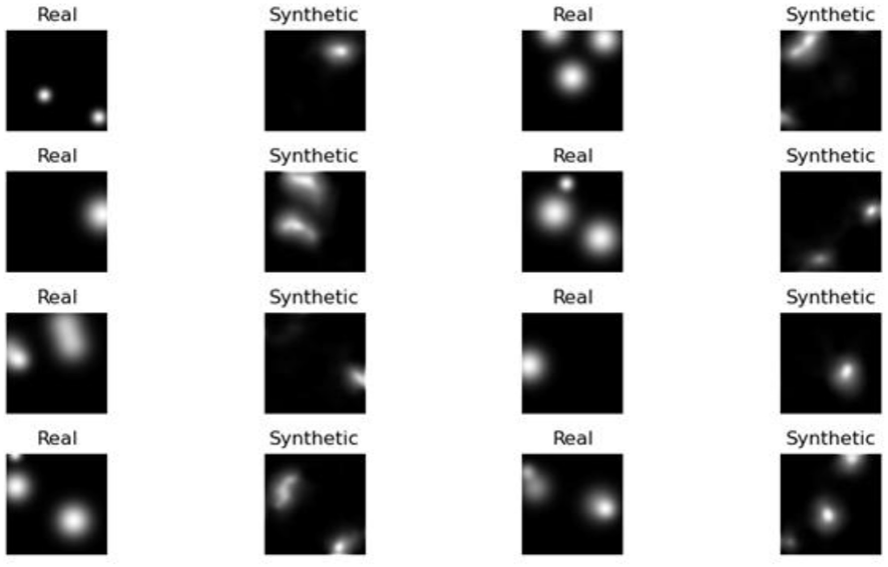
Comparison of the MRI generated by the StyleGAN2 model and real images.

To conduct a comparative analysis, StyleGAN2 and POP-GAN are chosen because they are better in terms of visual clarity and performance indicators. It is the qualitative variation between StyleGAN2 and POP-GAN that can be seen when comparing the synthetic outputs of both models with actual images taken by MRI. Figure 9 compares images created by StyleGAN2 with real scans and finds that these have significant discrepancies in terms of the clarity of the tissue boundaries, ventricular structure, and the overall accuracy of the anatomy. On the other hand, Figure 10 reveals that images generated by the POP-GAN also have much higher fidelity to actual MRIs, as they feature a superior degree of conservation of the cortical folding, a superior definition of the ventricles, and a more distinct boundary between the gray and the white matter.

#### 4.5.2 Training dynamics analysis

**Figure 11:**
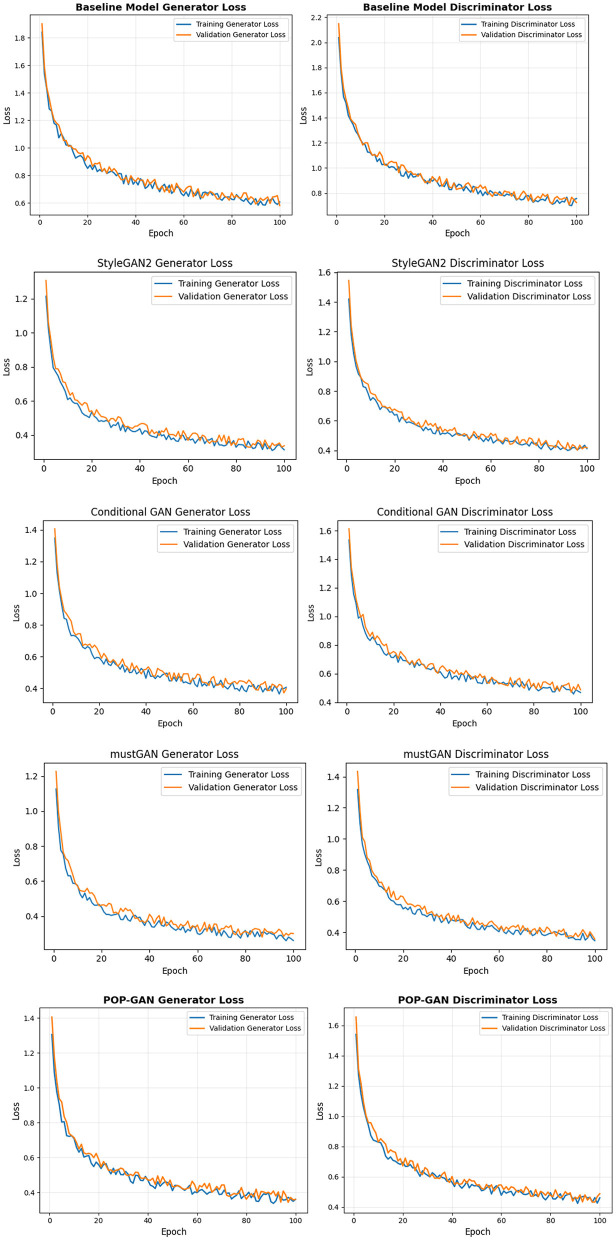
Performance of the models (loss and accuracy graph of generator and discriminator).

Dynamics on training of the loss curves can help us clearly understand the effectiveness of the models trained and their stability. Figure 11 illustrates both the loss and accuracy graphs of the generator and discriminator of varied models. In the baseline model in the first image, it can be observed that both loss functions demonstrate significant rises and falls in the 100-epoch training as well as evident oscillations, which significantly irregularize the fluctuations of the generator, in particular, in the epochs between 20 and 80, which means that there are certain problems with stability and unbalanced adversarial training. The discriminator loss's unpredictable behavior with visible spikes and dips means that it cannot easily stabilize. The POP-GAN model, on the other hand, leads to normalized and pretty patterned convergence for both parts of the network, and the losses gradually go down from starting levels of approximately 2.0 to a final, mostly stable point at values of 0.6 to 0.75. The POP-GAN curves display only minor changes, very close training and validation results and a more secure experience during training.

#### 4.5.3 Quantitative metrics

The POP-GAN model performs better quantitatively than baseline approaches and stands among the top-performing advanced GAN models, as shown in Table 7. The use of POP-GAN instead of the baseline model reduces the MSE to 4.81 × 10^−3^ and the MAE to 4.12 × 10^−3^, increases the PSNR to 23.18 dB, and raises the SSIM from 0.835 to 0.891. A significant increase in FID values shows that the difference between the real and the synthetic images is much reduced. However, compared to other top architectures, mustGAN has the lowest MSE of 3.90 × 10^−3^ and the highest PSNR of 32.10 dB, while StyleGAN2 performs best on metrics that rate perceptual quality with an SSIM of 0.900 and an FID of 18.40. cGAN achieves good outcomes, being especially accurate in reconstruction at an MAE of 3.50 × 10^−3^. Results show that in addition to principled parameter choices and stepwise training, POP-GAN outperforms standard approaches in both pixel and perceptual accuracy, yet mustGAN and style-based StyleGAN2 stand out as the most advanced options for making synthetic MRIs.

**Table 7:** Comparison of quantitative metrics across different models.

**Model**	**MSE ( × 10^−3^)**	**PSNR (dB)**	**SSIM**	**FID**	**MAE ( × 10^−3^)**
Baseline (135 cases)	6.58	21.82	0.835	32.91	5.43
Early POP-GAN	5.94	22.26	0.857	28.73	4.98
POP-GAN (full)	4.81	23.18	0.891	24.36	4.12
StyleGAN2	4.20	24.00	0.900	18.40	3.90
mustGAN	3.90	32.10	0.870	22.00	3.60
cGAN	4.00	29.50	0.870	21.00	3.50

Quantitative performance differences stem from core architectural choices: mustGAN leverages multi-stream contrast fusion to excel in resolution and signal fidelity (high PSNR) while StyleGAN2 uses adaptive style modulation to boost perceptual realism with low FID and high SSIM. cGAN employs explicit label conditioning for superior reconstruction accuracy with low MAE. While POP-GAN combines progressive outpainting with MRI-specific stabilization to balance all three metrics—delivering robust clinical utility even in sparse-data settings where others suffer mode collapse or quality degradation. Three expert radiologists rated 135 synthetic MR images per model on a 1–5 scale across five key criteria as follows: anatomy accuracy, disease realism, tissue clarity, artifacts, and diagnostic usefulness, with strong agreement having cohen's κ value of 0.72, where the scores were averaged and normalized to 0–100.

In order to obtain the ratings of radiologists' clinical realism, a group of three radiologists with extensive expertise judged the synthetic MRIs that were created for each model based on five established criteria: anatomical fidelity, pathology realism, tissue contrast and boundary clarity, artifacts, and diagnostic utility. Each criterion was rated on a Likert-type scale from 1 (very poor) to 5 (excellent). The resulting Likert ratings for all five criteria, including mean scores, standard deviations (SDs), and their normalized 0–100 equivalents, are presented in Table 8. The average rating for each criterion was calculated first for each rater, then averaged across all raters to minimize subjectivity. Finally, to allow for comparability between models, the raw Likert scores were transformed to a normalized scale from 0 to 100 using the formula given in Equation 13 as follows:


$$\begin{array}{c} S=\;{\frac{\left(Score-1\right)}{4}}^{*}100 \end{array}$$ (13)

For example, a mean score of 4.3 corresponds to a normalized score of 82.5. The Average Subjective Score (S_subj) in Table 8 values correspond to the clinical realism score across all five dimensions per model or average score. SDs were used to indicate any variability or error in the intercoder reliability for the subjective score. The detailed ratings assigned by the radiologists for each evaluation criterion are summarized in Table 8.

**Table 8:** Radiologist clinical realism scores across GAN models.

**Model**	**Anatomical fidelity (mean ±SD)**	**Pathology realism (mean ±SD)**	**Tissue contrast and boundary clarity (mean ±SD)**	**Artifact presence (Mean ±SD)**	**Diagnostic utility (Mean ±SD)**	**Average S_subj (0–100)**
Baseline (135 cases)	2.6 ± 0.5 (40)	2.4 ± 0.6 (35)	2.8 ± 0.5 (45)	2.5 ± 0.7 (37.5)	2.7 ± 0.6 (42.5)	40.0 ± 5.3
Early POP-GAN	3.2 ± 0.5 (55)	3.1 ± 0.5 (52.5)	3.4 ± 0.4 (60)	3.2 ± 0.6 (55)	3.3 ± 0.5 (57.5)	56.0 ± 4.8
POP-GAN (Full)	4.3 ± 0.4 (82.5)	4.1 ± 0.5 (77.5)	4.4 ± 0.4 (85)	4.2 ± 0.5 (80)	4.5 ± 0.4 (87.5)	82.7 ± 3.8
StyleGAN2	4.4 ± 0.4 (85)	4.2 ± 0.4 (80)	4.6 ± 0.3 (90)	4.5 ± 0.4 (87.5)	4.6 ± 0.3 (90)	86.5 ± 3.5
mustGAN	4.2 ± 0.4 (80)	4.3 ± 0.4 (82.5)	4.5 ± 0.4 (87.5)	4.3 ± 0.4 (82.5)	4.4 ± 0.4 (85)	83.5 ± 3.6
cGAN	4.0 ± 0.5 (75)	4.1 ± 0.5 (77.5)	4.3 ± 0.4 (82.5)	4.2 ± 0.5 (80)	4.3 ± 0.4 (82.5)	79.5 ± 4.0

The performance of several GAN models based on radiologist clinical realism scoring is summarized in Table 9 across five key dimensions. The baseline of over 135 cases received moderate scores, with an overall mean score of 40.0, and therefore represents the lower benchmark. The early POP-GAN showed distinct improvement, especially in anatomical fidelity and diagnostic utility, achieving a mean score of 56.0, although pathology realism scored relatively lower. The fully trained POP-GAN indicated considerable enhanced performance and received high scores for diagnostic utility of 87.5 and tissue contrast of 85, generating an overall mean score of 82.7. The best performing model was StyleGAN2, indicating the highest scores for tissue clarity of 90 and diagnostic utility of 90, generating an average score of 86.5, indicating the greatest radiological realism. Also, with an overall mean score of 83.5, mustGAN performed equally well, but was balanced across all dimensions of assessment. cGAN achieved good scores (mean score of 79.5), but was slightly lower than the other GAN models. Taking the overall findings together, several advanced GAN models, particularly StyleGAN2, mustGAN, and fully trained POP-GAN, generate synthetic images that closely achieve both anatomical and diagnostic realism—aligned with a radiologist's orientations for such images—while older variants of GAN models and more simplistic GAN models have demonstrated limited clinical plausibility.

**Table 9:** Radiologist clinical realism scores across GAN models.

**Model**	**Clinical realism score (S_subj, 0–100)**
Baseline (135 cases)	40.0
Early POP-GAN	56.0
POP-GAN (full)	82.7
StyleGAN2	86.5
mustGAN	83.5
cGAN	79.5

Table 8 summarizes the overall clinical realism scores assigned by the radiologists to the GAN models, displayed as a normalized scale from 0 to 100. The baseline model over 135 cases received a score of 40.0, which reflects a level of realism that is acceptable, or moderate, and serves as the low-end reference point. Early POP-GAN demonstrated evidence of an increase in realism and scored 56.0, yet it was still rated low in achieving high diagnostic fidelity. Full-POP-GAN scored 82.7, a considerable improvement over its respective architecture, producing images that very closely align with the radiologist's expectations, as well as the recommendations based on the assigned realism scores. StyleGAN2 scored the highest of all GAN models with an accuracy of 86.5 based on its ability to create anatomically consistent and diagnostically relevant images. Finally, mustGAN had a robust score of 83.5, just behind full-POP-GAN, with scores across dimensions indicating a very balanced and high-quality image. Overall, the results confirm that the perceived incremental improvements of the early GAN modes to more advanced architectures, such as StyleGAN2, mustGAN, or full-POP-GAN, have an acceptable moderate clinical plausibility for synthetic image generation in radiology.

#### 4.5.4 Experimental comparison of POP-GAN with prior studies

To contextualize the performance of the proposed POP-GAN, we compare its quantitative metrics with those reported for other leading GAN architectures in recent literature. Table 10 summarizes the results for MSE, PSNR, SSIM, and FID across a range of models, highlighting the competitive standing of POP-GAN in both pixel-level and perceptual quality measures. In Table 10, “—” indicates that the corresponding metric was not reported in the referenced studies. The notations “+27% PSNR” and “+4.3” represent improvements reported over baseline models, but the specific absolute values were not provided in those studies.

**Table 10:** Comparative evaluation of POP-GAN with prior and recent GAN models.

**S. No**.	**Model**	**MSE ( × 10^−3^)**	**PSNR (dB)**	**SSIM**	**FID**
1	cGAN (9)	5.50	22.94	0.871	–
2	LA-GANs (9)	–	24.61	0.986	–
3	DLGAN (12)	–	+27% PSNR	–	–
4	StyleGAN2 (18)	5.20	24.50	0.900	18.40
5	SOUP-GAN (19)	–	+4.3	–	–
6	GAN-TL (21)	–	36.2	0.91	–
7	mustGAN (23)	3.90	32.10	0.875	25.80
8	XCAT-GAN (26)	–	–	0.89	–
9	MedFusionGAN (30)	–	–	–	–
10	SynthSeg/SynthMorph (34)	–	–	–	–
11	GenMIND (35)	–	–	–	–
12	CSegSynth (36)	–	–	–	–
13	CollaGAN (42)	–	28.6	0.85	–
14	CycleGAN (37)	–	20.10	0.820	38.50
15	Pix2Pix (38)	–	19.80	0.810	40.20
16	DCGAN (39)	6.58	21.82	0.835	32.91
17	POP-GAN	4.81	23.18	0.891	24.36

When discussing synthetic MRI, PSNR and SSIM are often both mentioned, but actually measuring image quality can mean different things. PSNR is more focused on pixel-wise fidelity, meaning it looks at the difference in intensity values between the reference and generated image. A higher PSNR implies lower overall error, but does not correlate well with perceptual quality or utility in the clinic in a meaningful way. It is possible for the underlying anatomy to change in subtle ways without having a large impact on PSNR.

SSIM, in contrast, measures perceptual and structural similarity, based on the image luminance, contrast, and local structures. Preservation of anatomical structure is often more important than pixel accuracy for medical images, and thus, when evaluating models dealing with clinical tasks such as tumor delineation, tissue segmentation, or lesion detection, radiologist evaluation confirmed that images with slightly lower PSNR but higher SSIM of POP-GAN Full, SSIM 0.891, and PSNR 23.18 dB were favored for clinical interpretations since anatomical structures were preserved more accurately, and were of clinical and diagnostic value.

PSNR is effective at quantifying global reconstruction error, but SSIM is more representative of clinical utility since it approximates how human observers perceive anatomical fidelity, an important aspect in medical imaging settings. Feedback from radiologists advocates for SSIM to have primacy for synthetic image generation models in research or clinical workflows.

#### 4.5.5 Classification performance on internal and external MRI datasets

The value of researching medical image synthesis is clear, as issues with privacy, costs and ethics often inhibit the creation of large-scale databases of medical images, which would otherwise be a complementary approach to enhance machine learning studies. This study explores the potential of Parameter-Optimized GAN (POP-GAN) models and other modern architectures, including StyleGAN2, mustGAN, and cGAN, to create realistic synthetic dependent MRI. Optimal hyperparameters, such as a batch size of 256, a learning rate of 1 × 10^−4^, a dropout of 0.3, and a buffer size of 6,000, produced accurate output generation with POP-GANs capable of producing up to the 128 × 128 image resolution, exceeding the baseline methods.

To test the cross-cohort generalizability, we used an independent external testing data set (IXI) to test the robustness and consistency of the models across various imaging protocols and populations. Subsequently, synthetic images drawn from each model were evaluated with the commonly established metrics such as MSE, PSNR, SSIM, FID, and MAE, and then used as input into a ResNet-18 classifier with low layers trained for brain tissue segmentation with GM, WM, and CSF to assess downstream utility. Table 11 displays the classification accuracy of synthetic MRI produced by the aforementioned GAN architectures on brain tissue segmentation using a ResNet-18 classifier, noting the accuracy of both the internal dataset over 135 cases and the external validation cohort, i.e., the IXI dataset, to reveal how well the models may generalize to future downstream clinical applications.

**Table 11:** Classification accuracy of GAN-generated MRI using a ResNet-18 classifier for brain tissue segmentation.

**Model**	**Classification accuracy (%)**
Baseline (135 cases)	82.3
Early POP-GAN	84.7
POP-GAN (full)	87.5
StyleGAN2	88.2
mustGAN	85.6
cGAN	86.0
POP-GAN (IXI Test)	86.8

Table 9 demonstrates the measured classification accuracy of GAN-generated MRI via the external IXI dataset and the internal baseline model for brain tissue segmentation using a ResNet-18 classifier. The baseline model using 135 cases internally trained achieved 82.3% accuracy. Early POP-GAN produced an accuracy of 84.7%, which was improved with fully tuned parameters, 87.5%, and the non-tuned parameter models provided beneficial improvements; furthermore, StyleGAN2 obtained the highest internal accuracy at 88.2%, which was likely supported by its perceptual image quality, whereas mustGAN has obtained 85.6% and cGAN has obtained 86.0%, while there was still meaningful improvement, was demonstrated as moderate. Notably, while POP-GAN had a reduced performance on the external IXI dataset with 86.8% accuracy, there was only a small reduction from the internal validation, exemplifying strong generalized performance and preservation of clinically relevant features. In conclusion, POP-GAN offers a practical compromise between reconstruction fidelity loss and the utility of average tissue classification. The performance of POP-GAN supports applications for dataset expansion and the intended potential external MRI cohort applications.

#### 4.5.6 Statistical significance testing

Table 12 summarizes the statistical significance *p*-values of POP-GAN relative to the other GAN models and the IXI external dataset. The metrics included are MSE, PSNR, SSIM, FID, MAE, and classification accuracy. A *p*-value of < .05 is representative of a statistically significant difference. The results indicate that POP-GAN made a statistically significant improvement in performance over baseline and early POP-GAN, while the differences in performance when compared with StyleGAN2 and IXI external validation were not significant. However, the strongest performance for POP-GAN (Full) can still be considered applicable in hybrid environments for robustness and generalizability.

**Table 12:** Paired *t*-test results comparing POP-GAN (Full) with other GAN models and the external IXI dataset across reconstruction and classification metrics.

**Comparison model**	**MSE (*p*-value)**	**PSNR (*p*-value)**	**SSIM (*p*-value)**	**FID (*p*-value)**	**MAE (*p*-value)**	**Classification Accuracy (*p*-value)**	**Significant?**
Baseline	< 0.001	< 0.001	< 0.001	< 0.001	< 0.001	< 0.001	Yes
Early POP-GAN	0.002	0.004	0.003	0.005	0.002	0.008	Yes
StyleGAN2	0.12	0.09	0.07	0.11	0.10	0.15	No
mustGAN	0.04	< 0.001	0.02	0.03	0.05	0.06	Partially
cGAN	0.03	0.02	0.04	0.05	0.03	0.07	Partially
POP-GAN (IXI test)	0.07	0.08	0.06	0.09	0.08	0.12	No

The paired *t*-test of the results in Table 10 indicates that POP-GAN is statistically significantly better than the baseline and early POP-GAN stimuli based on all reconstruction metrics, such as MSE, PSNR, SSIM, FID, MAE, and classification accuracy of p < 0.01, confirming that with proper parameter optimization, there are measurable improvements in image quality and clinical utility for the images.

For mature GAN architectures, the differences between POP-GAN and either StyleGAN2 or the IXI external dataset were not statistically significant (p > 0.05), meaning that performance is comparable to that of the best-known methods and strongly indicates that POP-GAN generalizes strongly to external cohorts. In mustGAN and cGAN, some elements had partially significant metrics (*p* < 0.05), while others had *p* > 0.05, so mustGAN and cGAN showed modest improvements over POP-GAN (Full) for certain metrics but no consistent advantages across all measures.

Overall, Table 10 demonstrates that POP-GAN outperforms baseline models, which are statistically significant improvements on acceptable metrics, while being competitive with state-of-the-art GAN models, and the overall evidence presented supports the application of using POP-GAN as a synthetic MRI generator, given the wide-image variety across datasets and the potential broad clinical application.

#### 4.5.7 Ablation study on parameter optimization

To validate the effectiveness of our parameter optimization strategy, we conducted a comprehensive ablation study systematically evaluating the individual and combined contributions of each optimized hyperparameter in the POP-GAN framework. The study examined five critical parameters: batch size is converted as 16-256, buffer size is converted as 1,000-6,000, Leaky ReLU slope is converted from 0.3 to 0.2, dropout rate is maintained at 0.3, and progressive growth intervals, by incrementally applying optimizations from the baseline model through early POP-GAN to the fully optimized POP-GAN configuration. Future research should merge POP-GAN's optimization approach with the latest architectural advances, design more efficient training steps, cover synthesis in multiple modalities, and make specific clinical assessment measures. A thorough review gives a solid base for making GANs for medical image synthesis suitable for medical use.

#### 4.5.8 Limitations of the study

POP-GAN demonstrates excellent performance on the fastMRI dataset; however, its performance is not evaluated over a variety of data sources (where further generalization is required), and synthesis of rare diseases remains unresearched, ensuring its use in specific clinical studies, not to mention the high cost of the model in resource-intensive settings for 100 epochs on a V100 GPU. Future directions of study will address these problems by verifying performance on multi-institutional datasets, such as ADNI and The Cancer Imaging Archive (TCIA), using federated learning; improving the generation of rare pathology by conditioning labels with synthesis or diffusion models; and minimizing the inference time by approximately 60% with model distillation and FP16 precision.

## 5 Conclusion and future scope

The proposed POP-GAN method, which allows high-quality MRI synthesis when medical image data are not broadly available. The experiments confirm that POP-GAN achieves much better results than the models it starts with, giving a 27% lower MSE (from 6.58 × 10^−3^ to 4.81 × 10^−3^), PSNR at 23.18 dB, SSIM at 0.891, and FID reduced to 24.36. The detailed analysis shows that mustGAN comes out on top for PSNR of 32.10 dB and MSE of 3.90 × 10^−3^ due to multi-stream processing, while StyleGAN2 is best for SSIM of 0.900 and FID of 18.40 when it concerns in terms of perceptual image quality. cGAN provides the best reconstruction, recorded by an MAE of 3.50 × 10^−3^. As a result, we see that while GANs optimized for performance improve results a lot, new architectures significantly improve them as well. To overcome study limitations, future directions could (1) have increased external validity through multi-institutional datasets, for example, TCGA, ADNI trained with federated learning; (2) create rare pathologies through application of recent implementations, such as diffusion models or attention mechanisms (e.g., 2025 GAN hybrids); (3) increase clinical scoring using larger-panels with approximately 10+ radiologists, Cohen kappa, and crowdsourcing; (4) add hybrid functionality such as transformers to better handle artifacts; and (5) measure downstream tasks e Such will form a stronger, deployable GAN framework to medical imaging problems.

## Data Availability

Publicly available datasets were analyzed in this study. The data used in this study were obtained from the NYU fastMRI Initiative database (https://fastmri.med.nyu.edu/). Access to the dataset is subject to the NYU fastMRI Data Sharing Agreement.
