# Correction: Parameter-optimized generative adversarial network framework for synthetic MRI generation: fine-tuning critical variables for enhanced image fidelity

**DOI:** 10.3389/fmed.2026.1803906

**Published:** 2026-02-23

**Authors:** Anto Lourdu Xavier Raj Arockia Selvarathinam, Naveenkumar Anbalagan, Parvathaneni Naga Srinivasu, Jaeyoung Choi, Muhammad Fazal Ijaz

**Affiliations:** 1 Department of Data Science and Analytics, College of Computing, Grand Valley State University, Allendale, MI, United States; 2 Department of Information Technology, Sona College of Technology, Salem, Tamil Nadu, India; 3 Amrita School of Computing, Amrita Vishwa Vidyapeetham, Amaravati, Andhra Pradesh, India; 4 School of Computing, Gachon University, Seongnam-si, Republic of Korea; 5 Centre for Artificial Intelligence Research and Optimization (AIRO), Design and Creative Technology, Torrens University Australia, Melbourne, VIC, Australia

**Keywords:** conditional GAN, generative adversarial networks, medical image synthesis, mustGAN, parameter optimization, StyleGAN2, synthetic MRI generation

The Data Availability statement was erroneously given as “Publicly available datasets were analyzed in this study. This data can be found here: The dataset used in this study is publicly available from this link: https://arxiv.org/abs/2206.03671.” The correct Data Availability statement is “Publicly available datasets were analyzed in this study. The data used in this study were obtained from the NYU fastMRI Initiative database (https://fastmri.med.nyu.edu/). Access to the dataset is subject to the NYU fastMRI Data Sharing Agreement.”

The original version of this article has been updated.

